# Comparison of genome architecture at two stages of male germline cell differentiation in *Drosophila*

**DOI:** 10.1093/nar/gkac109

**Published:** 2022-02-15

**Authors:** Artem A Ilyin, Anna D Kononkova, Anastasia V Golova, Viktor V Shloma, Oxana M Olenkina, Valentina V Nenasheva, Yuri A Abramov, Alexei A Kotov, Daniil A Maksimov, Petr P Laktionov, Alexey V Pindyurin, Aleksandra A Galitsyna, Sergey V Ulianov, Ekaterina E Khrameeva, Mikhail S Gelfand, Stepan N Belyakin, Sergey V Razin, Yuri Y Shevelyov

**Affiliations:** Institute of Molecular Genetics of National Research Centre “Kurchatov Institute”, Moscow 123182, Russia; Skolkovo Institute of Science and Technology, Skolkovo 143026, Russia; A.A. Kharkevich Institute for Information Transmission Problems, Russian Academy of Sciences, Moscow 127051, Russia; Skolkovo Institute of Science and Technology, Skolkovo 143026, Russia; Institute of Molecular and Cellular Biology, Siberian Branch of Russian Academy of Sciences, Novosibirsk 630090, Russia; Institute of Molecular Genetics of National Research Centre “Kurchatov Institute”, Moscow 123182, Russia; Institute of Molecular Genetics of National Research Centre “Kurchatov Institute”, Moscow 123182, Russia; Institute of Molecular Genetics of National Research Centre “Kurchatov Institute”, Moscow 123182, Russia; Institute of Molecular Genetics of National Research Centre “Kurchatov Institute”, Moscow 123182, Russia; Institute of Molecular and Cellular Biology, Siberian Branch of Russian Academy of Sciences, Novosibirsk 630090, Russia; Institute of Molecular and Cellular Biology, Siberian Branch of Russian Academy of Sciences, Novosibirsk 630090, Russia; Novosibirsk State University, Novosibirsk 630090, Russia; Institute of Molecular and Cellular Biology, Siberian Branch of Russian Academy of Sciences, Novosibirsk 630090, Russia; Skolkovo Institute of Science and Technology, Skolkovo 143026, Russia; Institute of Gene Biology, Russian Academy of Sciences, Moscow119334, Russia; Faculty of Biology, M.V. Lomonosov Moscow State University, Moscow 119992, Russia; Skolkovo Institute of Science and Technology, Skolkovo 143026, Russia; Skolkovo Institute of Science and Technology, Skolkovo 143026, Russia; A.A. Kharkevich Institute for Information Transmission Problems, Russian Academy of Sciences, Moscow 127051, Russia; Institute of Molecular and Cellular Biology, Siberian Branch of Russian Academy of Sciences, Novosibirsk 630090, Russia; Novosibirsk State University, Novosibirsk 630090, Russia; Institute of Gene Biology, Russian Academy of Sciences, Moscow119334, Russia; Faculty of Biology, M.V. Lomonosov Moscow State University, Moscow 119992, Russia; Institute of Molecular Genetics of National Research Centre “Kurchatov Institute”, Moscow 123182, Russia

## Abstract

Eukaryotic chromosomes are spatially segregated into topologically associating domains (TADs). Some TADs are attached to the nuclear lamina (NL) through lamina-associated domains (LADs). Here, we identified LADs and TADs at two stages of *Drosophila* spermatogenesis – in *bam^Δ^^86^* mutant testes which is the commonly used model of spermatogonia (SpG) and in larval testes mainly filled with spermatocytes (SpCs). We found that initiation of SpC-specific transcription correlates with promoters’ detachment from the NL and with local spatial insulation of adjacent regions. However, this insulation does not result in the partitioning of inactive TADs into sub-TADs. We also revealed an increased contact frequency between SpC-specific genes in SpCs implying their *de novo* gathering into transcription factories. In addition, we uncovered the specific X chromosome organization in the male germline. In SpG and SpCs, a single X chromosome is stronger associated with the NL than autosomes. Nevertheless, active chromatin regions in the X chromosome interact with each other more frequently than in autosomes. Moreover, despite the absence of dosage compensation complex in the male germline, randomly inserted SpG-specific reporter is expressed higher in the X chromosome than in autosomes, thus evidencing that non-canonical dosage compensation operates in SpG.

## Graphical Abstract

**Graphical Abstract:**
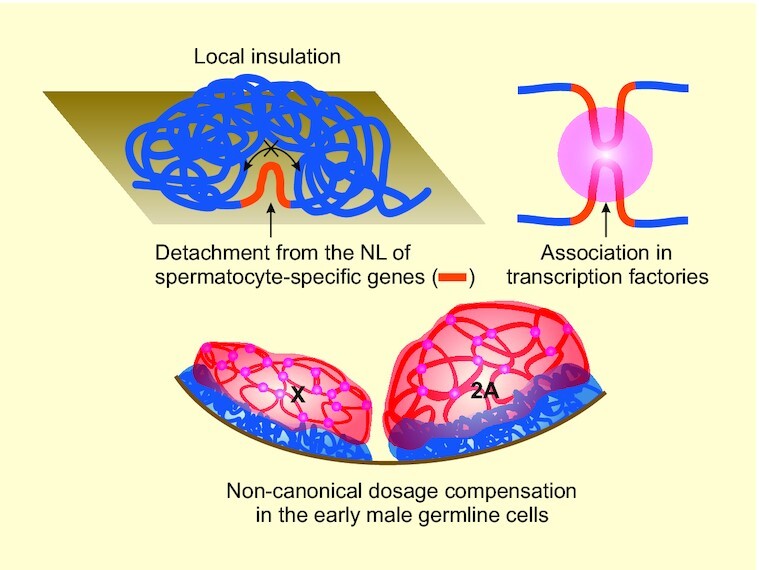
•1. Start of SpC-specific transcription correlates with detachment from the nuclear lamina and with spatial insulation. •2. SpC-specific genes are associated in transcription factories upon activation. •3. Non-canonical dosage compensation mechanism operates in the male germline.

## INTRODUCTION

Eukaryotic DNA must be densely packed to fit in a nucleus, but mechanisms of this packaging are still poorly understood. Using high throughput chromosome conformation capture (Hi-C) technique (1), topologically associating domains (TADs) were revealed as the units of chromosome folding (2–5). Super-resolution microscopy studies following Oligopaint labeling as well as single-cell Hi-C analysis have shown that TADs are separate chromatin globules with blurred boundaries in individual mammalian cells and with more precise boundaries in *Drosophila* cells (6–9). Recent Hi-C experiments with sub-kilobase resolution led to identification of many more TADs than were initially detected in both mammals and *Drosophila* (10–17). Furthermore, inter-TAD regions, which were revealed in *Drosophila* at 20-kb resolution (18), appeared to be comprised of a chain of short TADs, where each TAD corresponds to actively expressed gene or gene cluster (12,14).

Chromosome partitioning into TADs reflects more general phenomenon of spatial segregation of active (A) and inactive (B) chromatin compartments (1,12). The cohesin/CTCF-based chromatin loop extrusion process imposes an additional, dominant layer of regulation over compartmental segregation upon TAD formation in mammals (12,19–22). However, Rowley *et al.* (12) found no evidence that the same mechanism operates in *Drosophila*. Moreover, recent study has shown that CTCF contributes to the formation of not more than 10% of TAD boundaries in *Drosophila* central nervous system (23).

Phase separation was implicated to explain the segregation of active and inactive chromatin (24–26, reviewed in (27)). The basic principle of this segregation might be mediated by the ability of non-acetylated nucleosomes to aggregate with each other and by the absence of this ability in acetylated nucleosomes (18). Additionally, heterochromatin protein 1 (HP1) and linker histone H1 were shown to enhance formation of liquid droplets consisting of non-acetylated nucleosomes (28–30). In contrast, bromodomain-containing proteins stimulate the appearance of droplets consisting of acetylated nucleosomes (30).

TAD boundaries in *Drosophila* highly correlate with the presence of housekeeping genes (3,18,31–33). Apparently, permanently active chromatin of housekeeping genes has the intrinsic ability to fold into active TADs thus forming boundaries that delimit active and inactive chromatin. RNA polymerase II (Pol II) and cohesin were shown to participate in this process (16). However, it is still not fully understood what chromatin features are necessary and sufficient for the generation of new TAD boundaries in *Drosophila*. In particular, it was not systematically explored whether the initiation of tissue-specific transcription within inactive TADs leads to the emergence of active mini-TADs and to the partitioning of inactive TADs into sub-TADs.

In animal cells, chromosomes are attached to the nuclear lamina (NL), the layer beneath the inner nuclear membrane formed by lamins (which will be further referred to as Lam) and Lam-associated proteins (reviewed in (34)). Binding of chromosomes to the NL in lamina-associated domains (LADs) impacts chromatin three-dimensional (3D) organization (reviewed in (35)) by affecting both intra-TAD and inter-TAD interactions in mammals and *Drosophila* (36,37). LADs that were mapped in various cell types of *Drosophila*, mammals and nematode (38–42), contain silent or weakly-expressed genes, whereas ubiquitous and, to a lesser extent, tissue-specific genes (or at least their promoters) are detached from the NL (40,42–45). The displacement of individual activated loci from the NL to nuclear interior was repeatedly registered by fluorescence *in situ* hybridization (FISH) (reviewed in (46)). However, it remained unknown whether tissue-specific genes generally lose their association with the NL upon activation in *Drosophila*, because LADs were not previously mapped at consecutive developmental stages in this organism.

**Figure 1.**
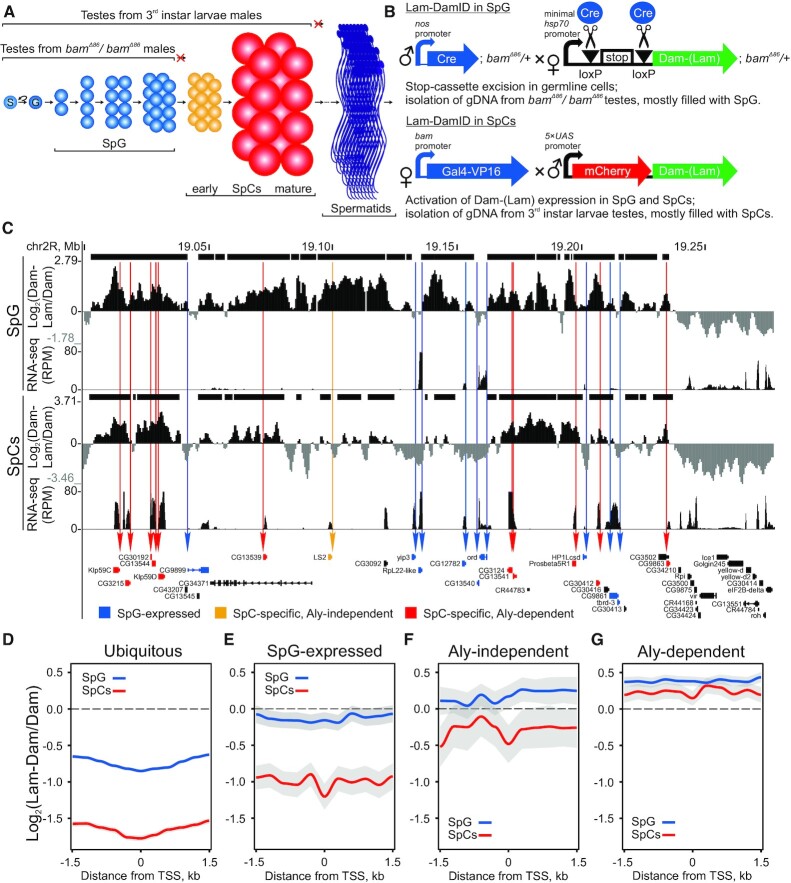
Ubiquitous and testis-specific promoters are detached from the NL upon activation. (**A**) Scheme of spermatogenesis from stem cell (S) to gonialblast (G), SpG, SpCs (early to mature) and spermatids (further steps of spermatid differentiation are omitted). Hub cells, somatic cyst cells as well as somatic sheath cells are not shown. (**B**) Principle of DamID in SpG and SpCs. (**C**) A screenshot of UCSC Genome Browser showing the log_2_(Dam-Lam/Dam) profiles as well as RNA-seq profiles (in RPM) in SpG and SpCs for the *59D* genome region of the 2R chromosome. HMM-determined LADs are indicated by black rectangles over DamID profiles. Arrows indicate the positions of promoters of testis-specific genes from the *59D* cluster. Color code is given below. (**D**–**G**) Averaged log_2_(Dam-Lam/Dam) profiles in SpG (blue curves) and SpCs (red curves) around TSSs of ubiquitous (**D**), SpG-expressed (**E**), Aly-independent (**F**), and Aly-dependent (**G**) genes.

*Drosophila* spermatogenesis (reviewed in (47)) is a useful model of cell differentiation (Figure 1A). During spermatogenesis, germinal stem cells adjoining to the somatic hub cells are self-renewed via asymmetric division, also producing a gonialblast. The latter undergoes four rounds of mitotic divisions resulting in 16-cell cysts filled with spermatogonia (SpG). Then, cells enter the meiotic prophase as spermatocytes (SpCs). During 3.5 days, the volume of SpCs increases 25-fold, and this growth is accompanied by transcription initiation of more than a thousand SpC-specific genes. This transcriptional burst is mediated by massive chromatin remodeling launched by *de novo* expression of testis-specific meiotic arrest complex (tMAC) as well as by testis-specific TBP-associated factors (TAFs) (48,49, reviewed in (50)). Transcription almost completely ceases just before meiosis, that results in 64 interconnected haploid spermatids. However, the stored transcripts are translated after meiosis, leading to gross alterations of spermatid morphology and, finally, to production of the motile sperm.

In somatic *Drosophila* tissues, two mechanisms likely mediate the equalization of expression of a single dose of X-linked genes in males with the double dose of X-linked genes in females or autosomal genes in males and females. The first mechanism relies on the activity of the canonical Male-Specific Lethal (MSL) dosage compensation complex which introduces H4K16 hyperacetylation in the male X chromosome thus making chromatin context more favorable for transcription (51–53, reviewed in (54)). However, depletion of MSL subunits in two *Drosophila* male cell lines resulted in only ∼1.4-fold decrease in X-linked expression (55,56). Moreover, X-linked transcription appears to be partially equalized in male and female *Drosophila* embryos during early developmental stages when the canonical dosage compensation system is not yet active (57). Thus, a second, yet unknown mechanism likely exists which leads to further ∼1.4-fold up-regulation of the X-linked genes in males (55). In male germline cells, several subunits of the MSL complex are not expressed and H4K16 acetylation is not enriched in the X chromosome compared to autosomes (58). Based on testis transcriptome analysis, various research groups have come to contradictory conclusions about the existence and magnitude of dosage compensation in *Drosophila* male germline cells (56,59–61). While Deng *et al.* (59) revealed a nearly complete equalization of X and autosomal gene expression, Shi *et al.* (60) found no evidence of dosage compensation, and two other groups (56,61) reported ∼1.5-fold enhanced expression of the X-linked genes.

Here, we performed a comprehensive analysis of chromatin organization, including identification of LADs, Hi-C, whole-chromosome FISH, RNA-seq, and transgene expression assay, to trace dynamic changes in genome architecture during male germline cell differentiation in *Drosophila*. In particular, we examined whether the initiation of tissue-specific transcription results in the detachment of chromatin from the NL and leads to the partitioning of large inactive TADs into smaller sub-TADs. We also explored whether the single X chromosome in SpG adopts the specific 3D configuration related to a non-canonical dosage compensation mechanism.

## MATERIALS AND METHODS

### Fly lines

Fly stocks were maintained under standard conditions at 25°C. Transgenic strains carrying *pUAST-attB-hsp70-loxP-stop-loxP-Dam*, *pUAST-attB-hsp70-loxP-stop-loxP-Dam-Lam*, *pUAST-attB-LT3-Dam* and *pUAST-attB-LT3-Dam-Lam* constructs were generated by *φC31*-mediated integration at the *attP40* site on the *2* chromosome in the *y, w*; *P{y[+t7.7] = CaryP}attP40*; *M{3xP3-RFP.attP}ZH-86Fb*; *M{vas-int.B}ZH-102D* line (62) as described earlier (63). The *nos-Cre* line was described previously (64). *pUAST-attB-hsp70-loxP-stop-loxP-Dam* or *pUAST-attB-hsp70-loxP-stop-loxP-Dam-Lam* constructs were combined with the *bam^Δ^^86^* mutation (65) by standard genetic crosses. *bam-Gal4-VP16* driver line (66) was kindly provided by Helen White-Cooper (Cardiff University). Random insertions of *P{CaSpeR-stil-βgal}* in different chromosomes were obtained by the use of *P*-element-mediated transformation in the *Df^(1^^)^w^67c23(2^^)^, y* embryos, performed by the standard protocol (67), and by further remobilization of transgene insertions. Insertions were mapped to definite chromosomes (Supplementary Table S1) and balanced using conventional genetic methods. The verification of single-copy insertion event in each transgenic line was carried out by qPCR with *lacZ* primers (Supplementary Table S2). In case of autosomal insertions, testes for the analysis of *lacZ* expression were isolated from adult males carrying hemizygous insertions in either paired or unpaired autosomes (in the latter case, normal chromosomes were opposed with the balancer chromosomes; Supplementary Table S1). To create the double RNAi knockdown of *Lam Dm0* and *Lam C* genes (hereafter Lam-KD), we first genetically combined *UAS-LamDm0-RNAi* (#107419; Vienna Drosophila Resource Center) and *UAS-LamC-RNAi* (#31621; Bloomington Drosophila Stock Center) hairpins in the common genotype. To obtain efficient knockdown of both *Lam* genes in SpCs, we crossed the resulting line with the *bam-Gal4-VP16* germline specific driver (66).

### Constructs

To generate *pUAST-attB-hsp70-loxP-stop-loxP-Dam-Lam* and *pUAST-attB-LT3-Dam-Lam* constructs, the ∼0.5-kb 5′-fragment of *Lam Dm0* ORF was PCR-amplified from *pNDamMyc-Lam* plasmid (kindly provided by Bas van Steensel) (68) using primers 5′-TAATGCGGCCGCTGTCGAGCAAATCCCGACGTGCTG and 5′-TGTTCCAGGTTCTTCCGCGTTTCCTC and digested with NotI and HindIII. Then, this fragment was cloned, together with the ∼1.6-kb HindIII-XbaI 3′-fragment of *Lam Dm0* ORF from the same plasmid, into NotI and XbaI sites of *pUAST-attB-hsp70-loxP-stop-loxP-Dam* or *pUAST-attB-LT3-NDam* vectors, respectively (64,69). The correct sequence of the resultant *pUAST-attB-hsp70-loxP-stop-loxP-Dam-Lam* and *pUAST-attB-LT3-Dam-Lam* constructs was verified by partial sequencing.

To generate the *P{CaSpeR-stil-βgal}* construct, the ∼0.6-kb fragment containing *stil* gene promoter was PCR-amplified from DNA of *Df^(1)^w^67c23(2)^, y* strain using primers 5′-GACTCTGCAGGCTGCCACCATGTTTCAGA and 5′-GCTCGGATCCATTTTTCAGTTTCATAAATGGG. The PCR product was first cloned into the *pGEM-T* easy vector (Promega), and then recloned by BamHI and PstI upstream from *lacZ* ORF into *P{CaSpeR-βgal}* vector (70).

### Lam-DamID

To carry out Lam-DamID specifically in SpG, we applied an earlier described tissue-specific DamID approach (64) for the *bam^Δ^^86^* testes (65) which is the commonly used model for analysis of mitotically dividing early germline cells mostly resembling SpG (71–74). Briefly, *pUAST-attB-hsp70-loxP-stop-loxP-Dam* and *pUAST-attB-hsp70-loxP-stop-loxP-Dam-Lam* constructs contain the removable stop-cassette, flanked by loxP sites, which blocks transcription from the basal *hsp70* promoter to the *Dam* or *Dam-Lam* ORFs (Figure 1B, upper panel). We genetically combined each of these constructs with the *bam^Δ^^86^* mutation. Next, we crossed the resultant lines with the *nos-Cre* line and isolated testes from the homozygous *bam^Δ^^86^* adult progeny. Since the *nos* (*nanos*) promoter is active only in the germline cells, elimination of the stop-cassette by Cre recombinase and subsequent genomic Dam or Dam-Lam methylation proceeds only in the germline cells of *bam^Δ^^86^* testes (i.e. mainly in SpG). The germline specificity of this procedure was validated by PCR-amplification of methylated fragments in the absence of *nos*-*Cre* (Supplementary Figure S1A, lane 3).

To perform Lam-DamID specifically in the SpCs, we employed an approach suggested by Southall *et al.* (69). In this approach, low levels of Dam and Dam-Lam expression, necessary for DamID, are achieved due to rare translation re-initiation events of *Dam* or *Dam-Lam* ORFs located downstream of the *mCherry* ORF in their common bicistronic mRNAs (Figure 1B, lower panel). To induce Dam or Dam-Lam expression in the germline, we crossed fly lines carrying *pUAST-attB-LT3-Dam* or *pUAST-attB-LT3-Dam-Lam* constructs with the *bam-Gal4-VP16* germline-specific driver (66). This driver expresses Gal4-VP16 activator beginning from the late SpG, with the peak of expression in early-mid primary SpCs (75). Then, testes from third instar larval males that are the progeny from these crosses were isolated. We used third instar larval testes, but not the testes from adult males since spermatogenesis in larvae does not proceed until the post-meiotic stages (76). Therefore, although larval testes contain a small fraction of SpG, they are mainly filled with early to mature SpCs, while, judging by mCherry fluorescence (Supplementary Figure S1B), Dam-Lam and Dam methylation is most pronounced in early-to-mid stage primary SpCs and declines in mature primary SpCs. The germline specificity of this procedure was validated by PCR-amplification of methylated fragments in the absence of *bam-Gal4-VP16* (Supplementary Figure S1C, lane 8).

After *in vivo* Dam-methylation, genomic DNA from either ∼200 *bam^Δ^^86^* testes from imago or ∼200 larval testes (each in two biological replicates) was isolated by DNeasy Blood and Tissue kit (Qiagen). The protocol for PCR-amplification of methylated genomic fragments (Supplementary Figure S1A, C) was performed as described previously (77), except that 16 cycles of PCR were applied for all the samples. Next, DamID products were sequenced on the Illumina Hi-Seq 2000 by Evrogen (www.evrogen.com) resulting in 41–52 million 100-nt single-end reads per sample (Supplementary Table S3).

### Hi-C library preparation

Hi-C experiments were performed on *bam^Δ^^86^*testes from imago that mostly contain SpG, as well as on third instar larvae testes that mostly contain SpCs. Experiments were performed in two and three biological replicates, respectively, as described previously (18), with minor modifications to the fixation and lysis steps. A total of 200–300 testes per replicate were fixed in PBS containing 2% formaldehyde for 10 min with occasional mixing. The reaction was quenched by the addition of 2 M glycine to a final concentration of 125 mM. Subsequently, testes were pelleted by centrifugation at 1000 × g for 10 min at 4°C, resuspended in 50 μl PBS, snap-frozen in liquid nitrogen and stored at −80°C. Testes were lysed in 1.5 ml isotonic buffer [50 mM Tris−HCl pH 8.0, 150 mM NaCl, 0.5% (v/v) NP-40 substitute, 1% (v/v) Triton X-100, 1 × Halt™ Protease Inhibitor Cocktail (Thermo Scientific)] on ice for 15 min after homogenization in a glass tissue grinder. Cells were pelleted by centrifugation at 2500 × g for 5 min, resuspended in 100 μl DpnII buffer (New England Biolabs), and pelleted again. All downstream steps were performed as described previously (18). Hi-C libraries were deep sequenced on the Illumina HiSeq 4000 and on the Illumina NovaSeq by GENEWIZ (USA; www.GENEWIZ.com) resulting in 23–144 million 100-nt or 150-nt paired-end reads per sample (Supplementary Table S3).

### 3C analysis

3C experiments were performed on chromatin isolated from *bam^Δ86^*imago testes or from third instar larvae testes in two biological replicates, as described previously (78), with some modifications. ∼300 testes per replicate were fixed and lysed as described just above. Next, cells were pelleted by centrifugation at 2500 × g for 5 min, resuspended in 100 μl NEBuffer 3 buffer (New England Biolabs), and pelleted again. Chromatin was treated with 0.3% SDS in NEBuffer 3, and DNA was digested by 500 U of NcoI (New England Biolabs) overnight. After enzyme inactivation at 65°C for 20 min, DNA ends were ligated by 75 U of T4 DNA ligase (Thermo Scientific) for 6 h. Next, formaldehyde cross-links were reversed at 65°C overnight in the presence of 300 μg Proteinase K (Sigma). DNA was extracted with phenol–chloroform and precipitated with ethanol.

Contact frequencies between several NcoI fragments and the anchor regions were analyzed by qPCR with TaqMan-probes (primers and TaqMan-probes are provided in Supplementary Table S2). The efficiency of PCR primers was estimated on the NcoI-digested and ligated pBAC RP98-22O15 DNA covering the *60D* region. Contact frequencies between the anchor region and the chosen fragments in *bam^Δ^^86^* or larval testes were normalized to that between the anchor region and the Reg1 fragment (the latter represents the most distant region among analyzed, located in another TAD).

### RNA-seq

To perform RNA-seq in control and Lam-KD larval testes, total RNA from ∼200 testes per replicate (in three biological replicates), was isolated from third instar larvae of *UAS-LamDm0-RNAi*/*bam-Gal4-VP16; UAS-LamC-RNAi/+* (Lam-KD) and *bam-Gal4-VP16* (control) males. RNA was isolated using Trizol (Invitrogen). RNA samples were subjected to poly(A)+ selection, cDNA synthesis and deep sequencing on Illumina NovaSeq in the GENEWIZ (USA; www.GENEWIZ.com), resulting in 33–40 million 150-nt paired-end reads per sample (Supplementary Table S3).

### Whole-chromosome FISH and image analysis

Microdissection libraries from individual polytene chromosomes from larval salivary glands of *Drosophila SuUR* mutant strain, in which heterochromatin was mostly not underreplicated (79), were prepared as described earlier (80) and kindly provided by Nikolay B. Rubtsov (Institute of Cytology and Genetics, SB RAS). For the whole-chromosome labeling, libraries from the X and 2L chromosomes were amplified by degenerate oligonucleotide-primed (DOP)-PCR in the presence of fluorescein-12-dUTP (Biosan) and tetramethyl-rhodamine-5-dUTP (Biosan), respectively.

The immuno-FISH procedure was performed according to Cremer *et al.* (81) with some modifications. Testes from 3–5-day old males were manually dissected in PBS and placed in drops of PBS on the poly-l-lysine-coated microscope slides. Then, testes were slightly squashed and fixed for 10 min at room temperature in PBS containing 4% paraformaldehyde. At the end of fixation, a few drops of 0.5% Triton X-100 in PBS were added and slides were washed three times for 3 min at room temperature in PBS containing 0.01% Triton X-100, and once for 15 min at room temperature in PBS containing 0.5% Triton X-100. Then, slides were incubated overnight in PBS with 20% glycerol. Further, the slides were processed six times as follows: slides were frozen in liquid nitrogen for 15–30 s, thawed at room temperature and soaked in PBS with 20% glycerol. Then slides were washed three times for 10 min at room temperature in PBS, incubated for 5 min in 0.1 M HCl, rinsed with 2× SSC and incubated for several days at 4°C in 2× SSC with 50% formamide. Slides were prehybridized with labeled X and 2L chromosome probes (0.5 μg each) at 42°C for 1 h in the buffer containing 50% formamide, 10% dextran sulfate, 0.2× SSC, 0.5 μg/μl salmon sperm DNA. Then, DNA was denatured by incubation at 85°C for 15 min, and slides were hybridized with the probes at 37°C for 3 days in a wet chamber. After hybridization, slides were consequently washed with 2× SSC for 30 min at room temperature, with 2× SSC for 30 min at 37°C, with 0.2× SSC for 30 min at 37°C, with 0.2× SSC for 30 min at 42°C and with 0.2× SSC for 10 min at room temperature. Then, slides were washed in PBST (0.1% Tween-20 in PBS) for 5 min at room temperature and incubated in the blocking solution (1% BSA in PBS) for 1 h at room temperature. After that, slides were incubated for 14 h at 4°C with ADL84 mouse monoclonal anti-Lam Dm0 antibody (82) (1:500; Developmental Studies Hybridoma Bank), washed with PBST three times for 15 min at room temperature and incubated for 2 h at room temperature with the secondary Alexa Fluor 647-conjugated donkey anti-mouse IgG antibodies (1:200; Abcam). Finally, slides were washed three times for 15 min at room temperature with PBST, mounted in Vectashield Antifade Mounting Medium (Vector Labs), covered with coverslips and sealed with nail polish.

Three-dimensional image stacks of the slides were recorded with a confocal Zeiss LSM 710 META laser scanning microscope (Zeiss). Optical sections with 0.4-μm intervals along the Z-axis were captured. Images were processed and analyzed by using *IMARIS* 7.4.2 software (Bitplane AG) with a blind experimental setup. Distances between the NL and the centers of masses (CMs) of chromosome territories (CTs) of X and 2L chromosomes were estimated as previously described (37). Briefly, we were unable to fully automatically reconstruct the surfaces of nuclei based on their Lam Dm0 immunostaining. Therefore, the nuclear rim of a particular nucleus was manually outlined in all optical sections of the stack by the middle of its Lam Dm0 staining to further reconstruct the surface of this nucleus automatically in *IMARIS*. The CT surfaces within the outlined nuclei were automatically reconstructed using manually adjusted thresholds separately for ‘red’ and ‘green’ channels. As a result, roughly estimated CT volumes were recorded. Then, CMs of CTs were automatically determined. To calculate the distance between CMs of CTs and the NL, the instrument ‘measurement point’ was positioned on the CM and another ‘measurement point’ was positioned on the reconstructed nuclear surface at the point of its earliest intersection with a progressively growing sphere of the first ‘measurement point’. The distance between two ‘measurement points’ (i.e. the shortest distance between the CM of CT and the middle of the NL) for X and 2L chromosomes in the corresponding nucleus was determined and recorded. In parallel, volumes of nuclei were retrieved and recorded, and the radii of nuclei were calculated assuming that nuclei are spherical. Finally, CM-NL distances were normalized to the nuclear radii. Data were obtained separately for SpG (i.e. germline cells of *bam^Δ^^86^* testes) and SpCs (which were selected for analysis in imago male testes by the criterion of large nucleus size) in two independent experiments with 50–75 nuclei per experiment (Supplementary Table S4).

### Immunostaining

Immunostaining was performed as previously described (42) with some modifications. Briefly, ∼50 testes from third instar larvae *UAS-LamDm0-RNAi*/*+; UAS-LamC-RNAi/bam-Gal4-VP16* males and from *bam-Gal4-VP16* control males were manually isolated in PBT (PBS containing 0.01% Tween-20) on ice and then rinsed in PBS. Testes were fixed in 4% formaldehyde (in PBT) for 30 min at room temperature. Then, testes were washed in PBT three times for 10 min at room temperature. Prior to staining, testes were incubated in PBTX (PBT containing 0.3% Triton X-100) with 0.3% sodium deoxycholate for 1 h, then in PBTX with 5% CELLect goat serum (MP Biomedicals) for 1 h. Testes were incubated with primary antibodies in PBTX containing 3% normal goat serum (NGS, Invitrogen) overnight at 4°C, washed in PBTX three times for 10 min at room temperature, incubated with secondary antibodies in PBTX containing 3% NGS overnight at 4°C, and then washed in PBTX three times for 10 min at room temperature in a dark chamber. Testes were stained with 4′,6-diamidino-2-phenylindole (DAPI, Sigma) in PBT for 10 min, and incubated in SlowFade^®^ Gold antifade reagent (Invitrogen) overnight at 4°C. As the primary, a mixture of murine monoclonal ADL67 and ADL84 anti-Lam Dm0 antibodies (82) (1:500; Developmental Studies Hybridoma Bank), and rabbit polyclonal anti-Lam C antibodies (83) (1:500; a gift from Paul Fisher) were used. As the secondary, Alexa Fluor 488-conjugated goat anti-mouse IgG (1:500; Invitrogen) and Alexa Fluor 647-conjugated goat anti-rabbit IgG (1:500; Invitrogen) were employed. DNA was counterstained with DAPI. Three-dimensional image stacks were recorded with a Zeiss LSM 510 META laser scanning confocal microscopy (Zeiss). To estimate the ratio of SpG, SpCs and somatic cells in *bam^Δ^^86^* mutant or 3^rd^ instar larvae testes, the germline cells were additionally stained with rat anti-Vasa antibodies (1:100; Developmental Studies Hybridoma Bank) and different cell types were counted in several images corresponding to the medial confocal slices of the whole testes.

### X-gal staining of testes

Manually-isolated testes from imago were fixed in 2% glutaraldehyde in PBS for 30 min, washed two times for 5 min in PBS, and stained with 0.24% 5-bromo-4-chloro-3-indolyl β-d-galactopyranoside (X-gal) at 37°C for 3 h in the buffer containing 0.15 M NaCl, 10 mM NaH_2_PO_4_ (pH 7.5), 1 mM MgCl_2_, 3.1 mM K_3_[Fe_II_(CN)_6_] and 3.1 mM K_4_[Fe_III_(CN)_6_], as it was previously described (84). Testes were stored at 4°C in 70% glycerol in PBT and then photographed using Leica MZ9.5 stereo zoom microscope with Leica DC300 digital camera (Leica Microsystems).

### RT-qPCR analysis

The real-time RT-qPCR assay for transgene expression was performed on cDNAs synthesized with random primers on total RNA isolated (in 3 biological replicates) from testes of hemizygous for *P{CaSpeR-stil-βgal}* transgene imago males, using SYBR Green chemistry and MiniOpticon Real-Time PCR System (Bio-Rad). Expression levels of β-galactosidase-encoding transgenes were determined using primers represented in Supplementary Table S2 and were normalized to *rp49* gene expression.

### Data analysis

#### Generation of lists of ubiquitously and tissue-specifically expressed genes

A gene was classified as ubiquitously expressed if it was expressed in a broad spectrum of tissues and organs. The following criteria were applied. (i) A gene should be represented by at least three (out of four) calls (i.e. values exceeding background) in the FlyAtlas RNA-chip database from each of 15 adult and larval *Drosophila* tissues/organs (GSE7763 (85)); (ii) at least one spliced transcript variant of this gene should be classified as ubiquitous by this criteria; and (iii) its transcripts per million (TPM) value should exceed 1 in both *bam^Δ^^86^* testes (GSE97182 (86)) and in control larval testes (our RNA-seq data). As a result, 4228 ubiquitously expressed genes were found (Supplementary Table S5). A gene was classified as testis-specific, if it was expressed only in testes. The following criteria were applied. (i) A gene should not be classified as ubiquitous; (ii) a gene should be represented by 4 calls in testes according to the FlyAtlas RNA-chip database (GSE7763 (85)); (iii) it should be represented by ≤3 calls in all other tissues/organs (except the fat body and the whole fly); (iv) its mean expression signal value in testes should exceed 20; (v) its mean expression signal value in testes should exceed that in the fat body; and (vi) according to our RNA-seq data, its TPM value in control larval testes should be ≥1, or if it was <1 TPM, then it should be ≥1 TPM in *bam^Δ^^86^* testes (GSE97182 (86)). As a result, 1286 testis-specific genes were found (Supplementary Table S5). Testis-specific gene was classified as starting to be transcribed in SpG (or at the earlier stages) if (i) it was classified as testis-specific; and (ii) according to RNA-seq data (GSE97182 (86)), its TPM value was >1 in *bam^Δ^^86^* testes. 97% of these genes had TPM values >1 in control larval testes and, thus, they likely continued to be active in SpCs. The remaining testis-specific genes were classified as starting to be transcribed in SpCs (i.e. SpC-specific). As a result, 272 testis-specific genes, expressed in SpG, as well as 1014 SpC-specific genes were found (Supplementary Table S5). A SpC-specific gene was classified as Aly-dependent if, according to RNA-chip data (GSE28728 (72)), its mean expression signal value in *aly*-mutant testes was more than two-fold lower than that in *yw* testes. The remaining SpC-specific genes, present in RNA-chip data from GSE28728, were classified as Aly-independent. As a result, 852 Aly-dependent and 135 Aly-independent SpC-specific genes were found (Supplementary Table S5). We note that many genes, which are expressed in somatic cells, utilize alternative SpC-specific promoters for their transcription in SpCs. These genes were not included in the list of SpC-specific genes because it is difficult to differentiate between their somatic and germline expression.

#### Lam-DamID analysis

Sequencing reads from two biological replicates of Dam and Dam-Lam samples were adapter clipped and uniquely mapped to the dm3/R5 genomic assembly by the *bowtie2* (87). Reads were counted by the *HTSeq-count* software (88) and mapped on 0.3-kb genomic bins. Read counts were merged between replicates, as they were highly correlated (Spearman's correlation coefficients ρ = 0.89–0.91; Supplementary Figure S2A). Dam-Lam signals were normalized to Dam-only signals, and log_2_(Dam-Lam/Dam) profiles were generated. LADs (Supplementary Table S6) were called with the two-state Hidden Markov Model (HMM) algorithm, as was described previously (42). Average log_2_(Dam-Lam/Dam) profiles around transcription start sites (TSSs) were generated using in-house scripts (available at GitHub https://github.com/foriin/Lam_Sperm) written in *RStudio IDE* using the R package as well as the *ggplot2*, *dplyr*, *GenomicRanges*, and *reshape2* packages. Briefly, log_2_(Dam-Lam/Dam) values were extracted and averaged for the 0.3-kb bins containing TSSs (i.e. for zero bins), as well as for four upstream and four downstream bins of the analyzed gene subsets, and then the resulting profiles were plotted.

#### Hi-C analysis

Sequencing reads from two biological replicates, mostly representing SpG, and from three biological replicates, mostly representing SpCs, were processed with the *distiller-nf* pipeline (version 0.3.3) (https://doi.org/10.5281/zenodo.3350926). Mapping was done on the chromosomes 2L, 2R, 3L, 3R, 4, X and M of the dm3/R5 *Drosophila* genome assembly with the default settings and with an option *no MAPQ filter*. For the analysis of the interaction matrix, the main diagonal and the second adjacent diagonal were removed. As two libraries had the read length of 150 nt, while the others had the read length of 100 nt, the 150-nt reads were trimmed to become uniform across all libraries. The trimming was done at the 3'-ends with *cutadapt* (http://journal.embnet.org/index.php/embnetjournal/article/view/200). Re-mapping of the trimmed libraries with the same approach did not result in substantial changes of mapping statistics, distributions of contact probabilities, and replicates similarities. Thus, the non-trimmed libraries were retained for further analysis. After that, only 2L, 2R, 3L, 3R and X chromosomes were considered. Hi-C maps were downsampled with the *cooltools* package (https://cooltools.readthedocs.io/en/latest/) to nearly the same total number of contacts. Then, the iterative correction (IC) (89) was applied, and samples were clustered with the R package *HiCRep* (90) based on the stratum-adjusted correlation coefficient (SCC). Clustering was performed on 10-kb Hi-C maps for bins separated by at most 10 Mb. SCCs obtained for each chromosome were averaged and used to build a hierarchical dendrogram (Supplementary Figure S2B). Next, replicates were merged with the *cooler* package (https://cooler.readthedocs.io/en/latest/), downsampled to nearly the same total counts, and corrected by IC, resulting in the final Hi-C maps for SpG and SpCs. TAD boundaries were called with the *Armatus* (version 2.2) software (91) (https://github.com/kingsfordgroup/armatus) on the log_2_-transformed IC Hi-C maps of the 5-kb resolution. To avoid boundary misidentification, regions of poor mappability were filled in with the mean contact frequency values of the nearest informative bins. Then *Armatus* was run with the parameter *γ* = 1.9 for SpG, or *γ* = 2.0 for SpCs (Supplementary Table S6).

Active (A) and inactive (B) compartments (1) were identified for 10-kb resolution IC Hi-C maps with the *cooltools* package. Positive first principal component (PC1) values corresponding to A compartment were selected by the correlation with gene expression. Next, for each chromosome, bins were ranked according to their PC1 values, and 1% of bins with the highest and lowest PC1 values were filtered out. Then, normalized (observed/expected) contact frequencies were averaged within quantiles of the PC1, and the resulting values were used for saddle plot generation. We defined the same number of PC1 quantiles for both A and B compartments across all chromosomes and stages to analyze changes of compartmentalization in SpCs relative to SpG, averaged by chromosomes.

The compartment strength, displaying the ratio of contact frequencies in the active or inactive compartments to that in the whole chromosome, was determined for 2-kb resolution IC Hi-C maps as described previously (24). Briefly, for each bin, the compartment strength was calculated as the mean normalized (observed/expected) frequency of contacts between this bin and other bins within the same compartment, divided by the mean normalized (observed/expected) frequency of contacts between this bin and all the bins in the chromosome.

The insulation score (IS) (92) was calculated for 2-kb resolution IC Hi-C maps using the *cooltools.insulation* module with 12-kb window size. Orientation of IS-curves around TSSs of 3′→5′ oriented genes was reversed, and then all IS-curves were smoothed using linear interpolation. To generate heat maps demonstrating the increased contact frequencies between TSSs of different classes of genes, mean contact frequencies between 2-kb bins containing TSSs (±10 bins around) and belonging to different TADs were averaged across the whole genome. The frequency of contacts between TSSs divided into transcription quantiles was calculated within the A compartment at 5-kb resolution.

The significance of differences between *P*_c_(*s*) curves for autosomes and the X chromosome was estimated as previously described (93). The analysis was performed on 20-kb resolution Hi-C maps. The median Hi-C signal of each diagonal was divided by the median Hi-C signal of the second diagonal in the Hi-C map. The median contact frequencies were plotted as a function of distance in double logarithmic (log_10_) coordinates. To determine the significance of differences in Hi-C signal decay, we approximated a part of *P*_c_(*s*) curve using linear model fitting (*lm* function from R) for values ranging from 4.6 to 5.7 in log-log scale. Then the difference of slope coefficient obtained on the previous step was calculated either between pairs of autosomes or between X chromosome and autosomes, and Wilcoxon rank-sum test was applied to these two groups.

#### RNA-seq analysis

Sequencing reads from three biological replicates of Lam-KD and control testes were adapter clipped, low quality reads with the length of less than 20 nt were filtered out and the remaining reads were uniquely mapped to dm3/R5 genome assembly. To test whether RNA-seq replicates were similar, sequencing reads were counted per 0.5-kb genomic bins. Then, counts were normalized for sequencing depth and clustering analysis was performed (Supplementary Figure S2C). Since replicates were highly correlated, they were merged, reads were counted for genes of BDGP5.78 annotation and converted to TPM values using the *Salmon* tool (94) (Supplementary Table S5). An analysis of resulting data was performed in *RStudio IDE* using the R packages *tximport*, *GenomicRanges*, *dplyr* and *ggplot2*. *Lam Dm0* and *Lam C* genes were excluded from analysis because they were the knockdown targets. Differentially expressed genes upon Lam-KD were identified by the *DESeq2* package for R (95) with the cutoff parameters *P* < 0.05 and TPM fold change >1.5 (Supplementary Table S5). RNA-seq data for *bam^Δ^^86^* and WT testes were retrieved from GSE97129 (86) and analyzed the same way as for Lam-KD and control testes (Supplementary Table S5).

### Statistical analysis

To estimate the non-random overlap between genes and domains, a permutation test with 10,000 permutations was employed, while the Mann–Whitney (M–W) *U*-test was applied to calculate *P*-values upon comparison of two distributions.

## RESULTS

### Aly-independent SpC-specific genes lose their association with the NL upon activation

To analyze changes in genome architecture during *Drosophila* spermatogenesis, we employed *bam^Δ^*^86^ (65) homozygous mutant testes from imago as well as wild-type testes from third instar larvae. *bam^Δ^^86^* mutant testes are the commonly used model for the analysis of mitotically dividing germline cells (71–73). The absence of Bam protein in these testes leads to the failure of mitotically dividing germline cells to differentiate into SpCs and, as a result, to the accumulation of cysts which are filled with the germline cells resembling SpG (74) (Figure 1A; Supplementary Figure S3A). In the testes of third instar larvae, spermatogenesis does not proceed until the post-meiotic stages (76). Therefore, germline cells in these testes, albeit containing a small fraction of SpG, are mainly represented by SpCs (Figure 1A; Supplementary Figure S3B). These SpCs may be not identical to the SpCs from adult males. However, according to our RNA-seq analysis, SpC-specific gene expression in larval testes highly correlates with that in imago testes (Supplementary Figure S4, Spearman's correlation coefficient ρ = 0.84, *P* < 10^–4^; RNA-seq data for imago testes were taken from Laktionov *et al.* (86)), thus indicating that basically the same mechanism is responsible for the activation of these genes in SpCs of both developmental stages. We note that isolation of pure material of SpG or SpCs using cell sorting is technically complicated because germline cells are enclosed by somatic cyst cells and are interconnected by ring canals in testes. Thus, an admixture of somatic and early germline cells was present in both sample types. However, SpG or SpCs represent the major fractions in the corresponding samples (Supplementary Figure S3C, D). For brevity, these samples will be further referred to as SpG or SpCs.

Using a tissue-specific DamID approach (64,69,96,97, reviewed in (98)), we identified chromosomal regions interacting with the Lam Dm0 (i.e. LADs) specifically in SpG of *bam^Δ^^86^* testes and in early-to-mid stage SpCs of third instar larvae testes (Figure 1B, C; see Materials and Methods for details). We revealed 12120 LADs in SpG with a median size of 2.7 kb, as well as 8698 LADs in SpCs with a median size of 3.6 kb (Supplementary Table S6). The actual LAD sizes are likely much larger because the HMM algorithm has not filled the gaps over genomic bins that contain zero mapped reads in the Dam profiles. We found that LADs cover 50.5% and 42.5% of the non-repetitive part of genome in SpG and SpCs, respectively. These results are consistent with published LAD coverage in other *Drosophila* organs and cell types (41,42).

The lower percentage of LAD coverage in SpCs compared to SpG may be partially caused by the transcriptional activation of numerous SpC-specific genes. In order to test this possibility, we compared the positions of transcriptionally active genes in SpG and SpCs with the layout of LADs at these developmental stages. RNA-seq data in *bam^Δ^^86^*testes (86) were employed to estimate expression in SpG (Supplementary Table S5). To analyze expression in SpCs, we performed RNA-seq analysis in third instar larvae testes (Supplementary Table S5). We also generated lists of ubiquitous and testis-specific genes (Supplementary Table S5; see Materials and Methods for details). Testis-specific genes were further classified according to the stage at which their transcription was initiated as starting to express in SpG or having the SpC-specific expression. Next, SpC-specific genes were subdivided into Aly-dependent and Aly-independent groups. The Aly protein, which is first expressed in early SpCs (99), is a major component of the tMAC complex (49), necessary for the second wave of activation of SpC-specific transcription at the later SpC stages (72,100). Therefore, Aly-independent SpC-specific genes are likely to be transcribed already in early SpCs (101), whereas Aly-dependent genes start to transcribe later, at mid or mature SpCs.

We visually analyzed the Lam-DamID profile in the *59D* region containing a cluster of 21 testis-specific genes (102). Nine testis-specific genes from this cluster start to express in SpG and continue expression in SpCs (marked by blue on Figure 1C). Seven of these genes are localized in the inter-LADs already in SpG, and all of them are localized in the inter-LADs in SpCs. Among twelve SpC-specific genes from the cluster, eleven genes are Aly-dependent (marked by red on Figure 1C). Surprisingly, despite active transcription in SpCs, all these Aly-dependent genes are localized in LADs in both SpG and SpCs. However, the single Aly-independent gene in this region (marked by orange on Figure 1C) is localized in a LAD in SpG and in an inter-LAD in SpCs. Another cluster of testis-specific genes from the *60D* region (102) contains seven SpC-specific genes (five genes are Aly-dependent and two genes are Aly-independent), and all these genes are localized in LADs in both SpG and SpCs (Supplementary Figure S5).

Next, we analyzed these trends across the entire genome. It is known that the detachment of active genes from the NL is most prominent at their TSSs (40,42–45). We generated heatmaps and averaged log_2_(Dam-Lam/Dam) profiles around TSSs of various groups of genes. As expected, in both SpG and SpCs, 93% of promoters of ubiquitous genes are localized in inter-LADs (*P* < 10^–4^ for their occasional colocalization, permutation test; Supplementary Table S5). Moreover, averaged log_2_(Dam-Lam/Dam) profile around promoters of ubiquitous genes has the saddle-like form with the minimum at the TSSs (Figure 1D, Supplementary Figure S6A). Similarly, 70% promoters of testis-specific genes, starting to express in SpG, are localized in the inter-LADs in SpG (*P* < 10^–4^, permutation test), and their averaged log_2_(Dam-Lam/Dam) profile around TSSs has negative values in both SpG and SpCs with a minimum at the TSSs in SpCs (Figure 1E, Supplementary Figure S6B). Negative values of averaged log_2_(Dam-Lam/Dam) profile with a prominent dip at the TSSs are also typical for Aly-independent (Figure 1F, Supplementary Figure S6C), but, surprisingly, not for Aly-dependent SpC-specific genes in SpCs (Figure 1G, Supplementary Figure S6D). Consistent with these observations, the presence of Aly-dependent gene promoters within inter-LADs in SpCs may be accidental (*P* = 1.0 for their occasional colocalization, permutation test), whereas the majority of Aly-independent promoters (67%; Supplementary Table S5) are highly non-randomly localized in the inter-LADs in SpCs (*P* = 0.008 for their occasional colocalization, permutation test). Finally, averaged Lam-DamID profiles around promoters of Aly-dependent and Aly-independent SpC-specific genes have positive values at the TSSs in neurons and embryonic Kc167 cells, where these genes are not expressed (Supplementary Figure S7; Lam-DamID data in Kc167 and neurons were taken from (41,42)). Altogether, these results indicate that initiation of transcription of at least Aly-independent SpC-specific genes correlates with the detachment of their promoters from the NL (but see Discussion about the Aly-dependent genes).

### SpC-specific genes increase topological insulation of neighboring regions and interact in nucleus space upon activation

**Figure 2.**
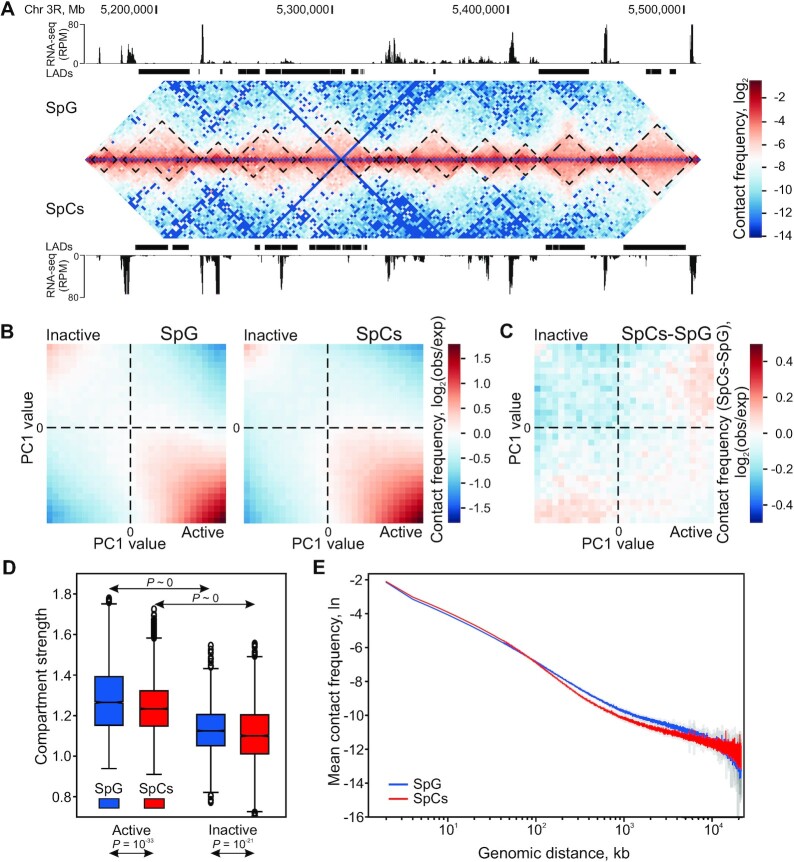
Compartmentalization is weakened upon transition from SpG to SpCs. (**A**) Hi-C heat map for the representative region of 3R chromosome in SpG (above the diagonal) and SpCs (below the diagonal). TADs (demarcated by black dotted lines), LADs (black rectangles) and RNA-seq profiles (in RPM, black peaks) in the corresponding region are indicated for SpG (above the Hi-C map) and SpCs (below the Hi-C map). (**B**) Saddle plots showing log_2_ values of contact enrichment for the intra-chromosomal contacts in all chromosomes as a function of their PC1 values in SpG (left panel) or SpCs (right panel). (**C**) Saddle plot showing subtraction of intra-chromosomal contact enrichment values in all chromosomes in SpCs from that in SpG. (**D**) Box plots showing compartment strength (see Materials and Methods for details) in the active and inactive compartments in SpG and SpCs. *P*-*v*alues were calculated using the M–W *U*-test. (**E**) *P*_c_(*s*) curves for SpG (blue curve) and SpCs (red curve).

To analyze changes in the 3D organization of chromatin during spermatogenesis, we generated Hi-C maps with 2-kb resolution in the *bam^Δ86^*mutant testes mostly containing SpG, and in the larval testes mostly containing SpCs. We identified 3206 TADs with a median length of 25 kb in SpG and 3274 TADs with a median length of 20 kb in SpCs (Figure 2A; Supplementary Table S6). TADs cover 93% and 95% of the genome in SpG and SpCs, respectively. 84–86% of TAD boundaries (±5 kb) are common between these two stages, while ∼15% of TAD boundaries appear or disappear in SpCs relative to SpG (Supplementary Figure S8).

As expected, the Hi-C analysis reveals the enrichment of intra-chromosomal contacts relative to inter-chromosomal contacts (Supplementary Figures S9 and S10), thus confirming the presence of CTs (reviewed in (103)) at both stages. In addition, inter-arm contacts appear to be more prominent within each chromosome than between chromosomes and are stronger in SpCs than in SpG (Supplementary Figures S9 and S10).

On a global scale, chromatin is spatially segregated into active and inactive compartments (1). Interestingly, like in embryos (5), and unlike in S2 cells (37), chromatin in SpG and SpCs is segregated into active, as well as into inactive compartments (Figure 2B; Supplementary Figure S11). 92% of active promoters in SpG and 88% in SpCs are in the active compartment, whereas LADs cover 68% and 57% of the inactive compartment in SpG and SpCs, respectively. Upon transition from SpG to SpCs, the strength (24) of active and inactive compartments, characterizing their spatial isolation, is notably weakened (Figure 2C, D). The *P*_c_(*s*) curves representing the decay of contact frequency as a function of genomic distance *s*, also demonstrate that distant interactions become weaker in SpCs (Figure 2E). Given the enhanced inter-arm contacts in SpCs relative to SpG (Supplementary Figures S9 and S10), these results indicate a stronger intermingling of chromatin within CTs in SpC than in SpG. This may be due to the presence of enlarged CTs at the SpC stage (Supplementary Figure S12).

**Figure 3.**
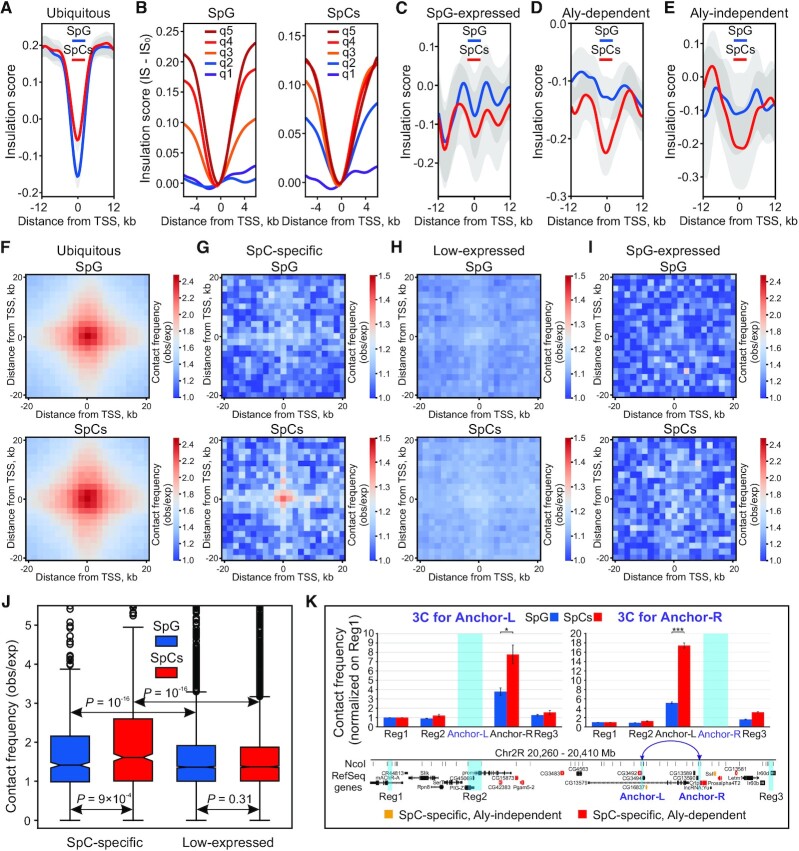
Ubiquitous and SpC-specific genes increase topological insulation of neighboring regions and interact in the nuclear space. (**A**, **C**–**E**) Averaged IS profiles in SpG (blue curve) and SpCs (red curve) around the bin carrying promoters of ubiquitously expressed (**A**), expressed in SpG testis-specific (**C**), Aly-dependent SpC-specific (**D**) or Aly-independent SpC-specific (**E**) genes. Only genes without any other expressed TSSs in SpG and SpCs within 12 kb from their TSSs were taken for this analysis. IS profiles were calculated for 2-kb resolution Hi-C heat maps using 12-kb sliding window. (**B**) Averaged IS profiles around the bin carrying promoters minus averaged IS values in the zero bin (IS_0_) for all the genes divided into 5 quantiles according to the total expression in the zero bin (where q1 corresponds to low expression and q5 – to high expression) in SpG (left panel) and SpCs (right panel). (**F**–**I**) Averaged contact frequency heat maps (observed/expected) around bins, carrying ubiquitous (**F**), SpC-specific (**G**), low-expressed (TPM < 1) (**H**) or testis-specific expressed in SpG (**I**) gene promoters, in SpG (upper panels) or in SpCs (lower panels). (**J**) Box plots showing fold change of contact frequencies (observed/expected) between SpC-specific or low-expressed (i.e. with TPM < 1) gene promoters located within the same TADs in SpG (blue) and SpCs (red). *P*-*v*alues were calculated using the M–W *U*-test. (**K**) qPCR analysis of 3C-determined contact frequencies at the *60D* region. The relative contact frequencies between the anchor points (Anchor-L on the left panel or Anchor-R on the right panel) and Reg1, Reg2, Anchor-L, Anchor-R or Reg3 regions, normalized on contact frequency between the corresponding anchor and Reg1, is plotted for SpG (blue columns) and SpCs (red columns). A genomic map from UCSC Browser of the analyzed region with the indication of Refseq genes and NcoI sites is shown below the plots. Aly-dependent and Aly-independent SpC-specific genes are marked by orange and red, respectively. *P*-values < 0.05 (*) and <10^–3^ (***) were calculated using the Student's two-tailed *t*-test.

We next explored whether the initiation of SpC-specific transcription within inactive TADs leads to the partitioning of these TADs into sub-TADs. Since SpC-specific genes are rather small (median size is 1.3 kb; Supplementary Table S5), we were unable to detect *de novo* emerged active mini-TADs harboring transcribed SpC-specific genes (if these TADs were formed) at the current Hi-C resolution. However, active chromatin of SpC-specific genes is expected to suppress contacts between the nearby inactive chromatin regions. In agreement with numerous published observations (3,18,31–33), we revealed highly non-random presence of ubiquitously expressed housekeeping genes at TAD boundaries in SpG and SpCs (*P* < 10^–4^ in both cases, permutation test). Consistent with these findings, the averaged IS profile (92), characterizing the strength of insulation (the lower the IS, the stronger the insulation), sharply declines at the promoters of ubiquitous genes (Figure 3A; Supplementary Figure S13A). In general, it appears that the higher total expression in a bin, the stronger the dip in the IS profile (Spearman's correlation coefficients for SpG and SpCs are equal to 0.89 and 0.99, *P* = 0.037 and *P* = 10^–24^, respectively; Figure 3B). Importantly, the dip in the averaged IS profile is also present at testis-specific genes starting to express in SpG (Figure 3C; Supplementary Figure S13B), as well as at both Aly-dependent (Figure 3D; Supplementary Figure S13C) and Aly-independent (Figure 3E; Supplementary Figure S13D) SpC-specific genes. Moreover, the initiation of SpC-specific transcription correlates with the transition of some SpC-specific genes from inactive compartment in SpG to active compartment in SpCs (Supplementary Figure S13E).

If the activity of SpC-specific gene promoters results in the partitioning of TADs into sub-TADs, one would expect the non-random colocalization of the majority of these promoters with the newly arisen TAD boundaries in SpCs. However, only 10% of SpC-specific gene promoters in SpCs are positioned within 2.5-kb distance from the TAD boundaries, not associated with the ubiquitous genes (Supplementary Figure S14; *P* = 0.45 for their occasional colocalization, permutation test), and only 5.2% of SpC-specific gene promoters are positioned in close vicinity to the newly arisen TAD boundaries in SpCs (*P* = 0.65 for their occasional colocalization, permutation test). Therefore, our analysis indicates an accidental colocalization of SpC-specific gene promoters with both types of TAD boundaries. Based on these results, we conclude that although initiation of SpC-specific transcription within inactive TADs may lead to local spatial insulation and chromatin transition from inactive to active compartment, it does not result in the partitioning of these TADs into sub-TADs.

Recent findings indicate that housekeeping genes frequently interact with each other in 3D nucleus space (12,33). Consistent with these results, our Hi-C data revealed a strong association between ubiquitously expressed genes in both SpG and SpCs (*P* < 10^–40^, M–W *U*-test; Figure 3F). We next examined whether such association is inherent for SpC-specific genes. We found, that across the whole genome, SpC-specific genes interact with each other more frequently in SpCs than in SpG (*P* = 10^–39^, M–W *U*-test; Figure 3G). At the same time, contact frequency between weakly-expressed or silent genes (i.e. with TPM < 1) remains low and is not notably altered in SpCs relative to SpG (*P* = 0.8, M–W *U*-test; Figure 3H). In contrast with SpC-specific genes, both in SpG and in SpCs, we did not detect an elevated association between testis-specific genes starting to express in SpG (*P* = 0.6, M–W *U*-test; Figure 3I). Importantly, an increased contact frequency in SpCs was observed between SpC-specific genes located within the same TADs (Figure 3J). We confirmed an increased association between SpC-specific genes in SpCs by performing 3C experiments with two SpC-specific genes located within a shared TAD from the *60D* region. When an anchor (designated Anchor-L in Figure 3K) was set at the SpC-specific gene *CG3492*, its contact frequency with SpC-specific gene *Crtp* (designated Anchor-R in Figure 3K) was notably higher in SpCs than in SpG. When an anchor was reciprocally set at the *Crtp* gene, the contact frequency between these two genes was similarly increased in SpCs compared to SpG (Figure 3K). At the same time, the contact frequency of Anchor-R with Reg3, which is nearly at the same distance as Anchor-L, was low in both SpG and SpCs. Collectively, these data point to *de novo* gathering of SpC-specific genes in gene hubs or transcription factories (reviewed in (104–106)) at the SpC stage when they become activated.

### In SpG and SpCs, X chromosome is stronger bound to the NL than autosomes

**Figure 4.**
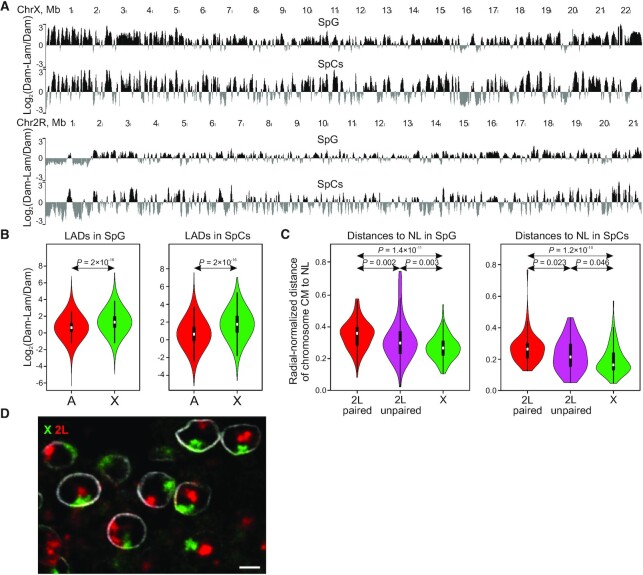
In SpG and SpCs, the X chromosome demonstrates stronger binding to the NL than autosomes. (**A**) A screenshot of UCSC Genome Browser showing the log_2_(Dam-Lam/Dam) profiles for the whole X and 2R chromosomes in SpG and SpCs. (**B**) Violin plots showing distributions of log_2_(Dam-Lam/Dam) values on the X chromosome and on autosomes in SpG and SpCs. *P*-*v*alues were calculated using the M-W U-test. (**C**) Violin plots showing radial-normalized distances from the CMs of the paired or unpaired 2L CTs or of the single X CTs to the NL in SpG (in *bam^Δ^^86^*-mutant testes) and in SpCs (in WT testes). *P*-*v*alues were calculated using the M-W *U*-test. (**D**) Single confocal slice image of FISH probes corresponding to the euchromatic arm of the X chromosome (green) and of the 2L chromosome (red) in *bam^Δ^^86^*-mutant testis. NL is visualized by immunostaining with anti-Lam Dm0 antibodies (white). Scale bar 3 μm.

Interestingly, in both SpG and SpCs, the Lam-DamID profile is higher in the X chromosome than in autosomes (Figure 4A). Quantitative evaluation shows that, in SpG and SpCs, the median log_2_(Dam-Lam/Dam) values for the whole X chromosome are 1.4-fold and 1.7-fold higher than for autosomes, respectively. This effect is even more pronounced in LADs (1.6- and 2.2-fold higher in SpG and SpCs, respectively; Figure 4B). Therefore in SpG, the single X chromosome is more tightly bound to the NL than pairs of autosomal homologues, and this preferential binding is further increased in SpCs.

To examine the spatial proximity of the X chromosome and autosomes to the NL during spermatogenesis, we performed dual-color FISH using probes corresponding to the euchromatic portions of the X and 2L chromosomes (see Materials and Methods for details). NL was visualized with anti-Lam immunostaining. We found that, in the majority of nuclei, 2L homologues were paired, thus forming common CTs. Unpaired homologues with separate CTs were detected in only ∼7% of nuclei. Using *IMARIS* software, we quantified the distances from the CMs of the X or 2L CTs (separately for the paired and unpaired 2L homologues) to the NL in both SpG (*bam^Δ^^86^* testes) and SpCs (WT testes) (Supplementary Figure S15) and normalized these distances on the nuclear radii (Supplementary Table S4). In agreement with early cytological observations showing a pronounced peripheral localization of chromosomes in maturing SpCs (107), we also revealed that in SpCs, both X and 2L chromosomes occupy a more proximal radial position to the NL than in SpG (Figure 4C). This peripheral localization is detected despite the enlarged volumes of X and 2L CTs in SpCs compared to SpG (Supplementary Figure S12). Importantly, at this overall disposition, the median value of radial-normalized distances from the X chromosome to the NL in SpG appears to be 1.4-fold less than that of paired 2L homologues (Figure 4C, D). The unpaired 2L homologues mostly demonstrate intermediate values (Figure 4C, left panel). In SpCs, the proximity of the X chromosome to the NL is even more prominent (1.8-fold closer) compared to the paired 2L homologues, whereas the unpaired homologues also occupy an intermediate position (Figure 4C, right panel). Collectively, our findings imply that, in *Drosophila* male germline cells, the single X chromosome is in more intimate association with the NL than either paired or unpaired autosomes.

### SpC-specific genes are mostly down-regulated upon Lam-KD in SpCs

**Figure 5.**
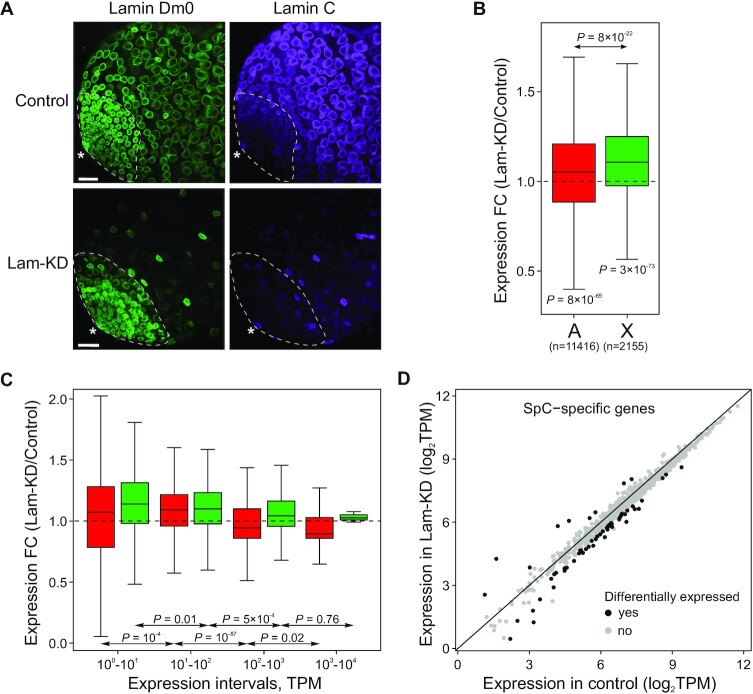
Upon Lam-KD in SpCs, low-expressed genes mostly increase their expression, while actively expressed genes mostly decrease their expression. (**A**) Immunostaining of third instar larvae testes from control (upper panel) or *LamDm0* RNAi; *bam-Gal4-VP16/LamC* RNAi (Lam-KD) (lower panel) males with anti-Lam Dm0 (green) and anti-Lam C (violet) antibodies showing that both lamins are effectively depleted upon Lam-KD in SpCs. Scale bar 30 μm. Testis tip is indicated by asterisk. Approximate zone of SpG is outlined by dotted line. Cells located further from the tip, which retain both lamins upon Lam-KD, have somatic origin. (**B**, **C**) Fold change of expression (according to RNA-seq data) of autosomal (red) and X-chromosomal (green) genes upon Lam-KD in germline cells of larvae testes. Genes were divided into four groups according to their expression in control cells (**C**). In (**B**, **C**), only genes whose TPM values in control testes exceed 1 were considered. *P*-*v*alues were calculated using the M–W *U*-test. (**D**) Scatter plot showing expression changes of SpC-specific genes in Lam-KD and control larval testes. Differentially expressed SpC-specific genes (i.e. genes with fold change (in TPM) > 1.5 and *P* < 0.05) are indicated by black dots.

Next, we explored whether increased binding of the X chromosome to the NL results in stronger repression of X-linked genes. If this were the case, one would expect to observe preferential activation of X-linked genes compared to autosomal genes upon the release of chromosomes from the NL. As was shown previously, RNAi knockdown of *Lam Dm0* in S2 cells resulted in the removal of total chromatin from the NE (37). Unfortunately, we failed to obtain an efficient RNAi knockdown of *Lam Dm0* in *bam^Δ^^86^* mutant testes using *nos-Gal4* germline-specific driver. However, we succeeded in performing an efficient double RNAi knockdown of *Lam Dm0* and *Lam C* genes in SpCs of larval testes. This was achieved by employing a *bam-Gal4-VP16* germline specific driver (66,75) (Figure 5A).

RNA-seq analysis has shown that, upon Lam-KD in SpCs, expression of both autosomal and X-linked genes, which have transcripts per million (TPM) values > 1 in control larval testes, is slightly enhanced (Figure 5B). Importantly, we observed that X-linked genes indeed become more up-regulated than autosomal genes under these conditions (Figure 5B; Supplementary Table S5). Interestingly, this up-regulation mainly involves weakly and moderately expressed genes. In contrast, highly active autosomal genes were even slightly down-regulated (Figure 5C). Minor variations in the number of somatic cells in testes, including a small fraction of the fat body co-isolated with larval testes, may differently affect RNA levels of various groups of genes. Thus, we analyzed whether the observed effects are also inherent for SpC-specific genes which are actively expressed exclusively in SpCs (the median expression in control larval testes is 145 TPM; Supplementary Table S5). We found that, upon Lam-KD, SpC-specific genes are mostly down-regulated (median Lam-KD/control ratio 0.89, *P* = 10^–4^, Wilcoxon signed-rank test; Figure 5D). This trend is especially evident when only differentially expressed SpC-specific genes are considered (designated by black dots in Figure 5D). We conclude that the NL exerts a faint suppressive effect on the weakly expressed genes in SpCs, and that this effect is stronger for X-linked than for autosomal genes. At the same time, NL integrity ensures a normal expression level of actively expressed genes (including SpC-specific genes).

### In SpG, the active compartment is more ‘open’ and more spatially segregated from the inactive one in the X chromosome than in autosomes

Although several components of the MSL complex are not expressed in the germline cells of *Drosophila* males (58), RNA-seq analysis has revealed partial equalization of X-linked and autosomal gene expression (X/2A = 0.9) in *bam^Δ^^86^* mutant testes (56,59). Based on the RNA-seq data from Laktionov *et al.* (86), we also found that in *bam^Δ^^86^* mutant testes, the X/2A median expression ratio for the expressed euchromatic genes (with TPM > 1) is 0.75 (Supplementary Table S5). Without any equalization, an 0.5 X/2A ratio is expected. Of note, this 1.5-fold enhanced expression of X-linked genes in early germline cells may be slightly overestimated because *bam^Δ^^86^* mutant testes also contain a fraction (∼36%, Supplementary Figure S3C) of somatic cells where 2-fold up-regulation of X-linked genes is maintained. With this correction, median expression of X-linked genes in SpG appears to be ∼1.2-fold higher than that of autosomal genes.

**Figure 6.**
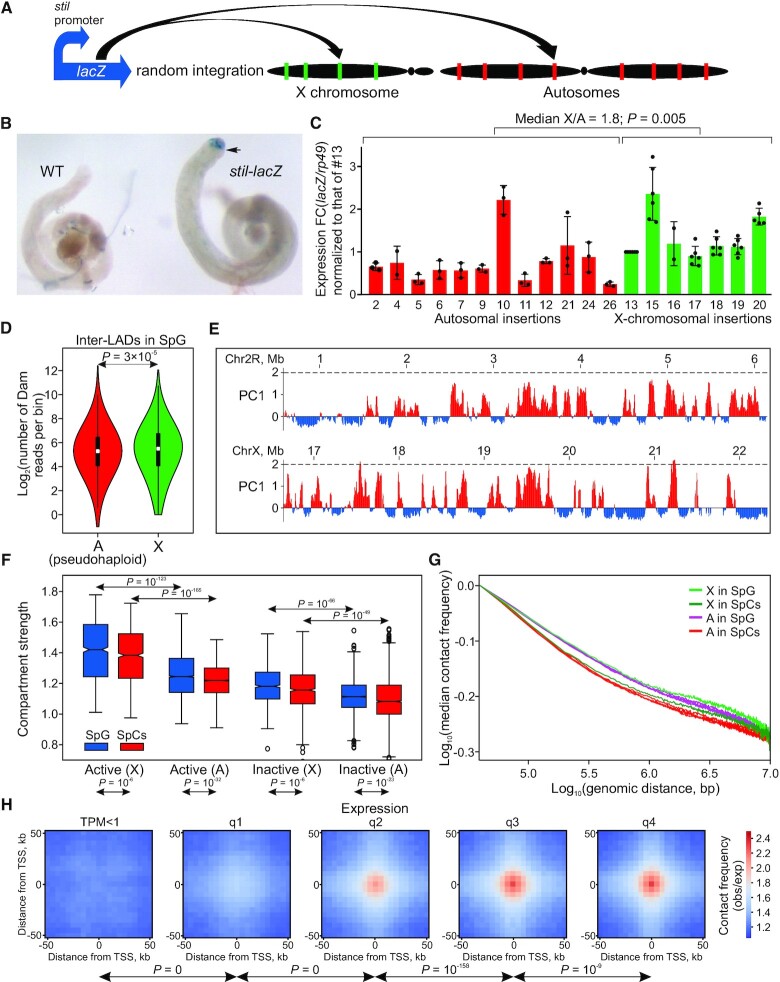
In SpG, active chromatin is more ‘open’, compartmentalized and favorable for transcription in the X chromosome than in autosomes. (**A**) Principle of the transgene-based assay. (**B**) X-gal staining (blue) of control (WT) testes or testes carrying the *stil-lacZ* transgene. Zone of SpG and early SpCs is indicated by an arrow. Slide was captured with the optical magnification ×60. (**C**) RT-qPCR analysis of *lacZ* expression in testes with autosomal or X-chromosomal insertions of the *stil-lacZ* transgene. *rp49* expression was used for within sample normalization. Only lines carrying one dose of transgene were compared. Autosomes carrying transgene were in the unpaired state. Relative expression of each transgene was normalized to the relative expression of line #13. Average values ± SD from two to six independent experiments are shown. M–W *U*-test was used for comparison of autosomal and X-chromosomal expression. (**D**) Violin plots showing log_2_ distribution of the number of Dam reads per 300-bp bin in the inter-LADs of SpG for pseudohaploid autosomes (red) and the X chromosome (green). White rectangles indicate median values. *P*-*v*alues were calculated using the M–W *U*-test. (**E**) PC1 profiles for the representative regions of 2R chromosome (upper panel) and of the X chromosome (lower panel). (**F**) Box plots showing compartment strength in the active and inactive compartments separately for the X chromosome (X) and autosomes (A) in SpG and SpCs. *P*-*v*alues were calculated using the M–W *U*-test. (**G**) *P*_c_(*s*) curves at mid- and long-range genomic distances separately for each autosome in SpG (purple curves), or for the X chromosome in SpG (light green curve), for each autosome in SpCs (red curves) or for the X chromosome in SpCs (dark green curve). (**H**) Averaged Hi-C heat maps in SpG around bins carrying TSSs of genes grouped by quantiles of expression in SpG (in *bam^Δ^^86^*-mutant testes). *P*-*v*alues were calculated using the M-W U-test.

To test by an alternative approach whether there is X chromosome-specific gene activation in the male germline cells, we utilized a *lacZ* reporter gene directed by a germline-specific *stil* promoter (108). This reporter gene was randomly integrated at different chromosomal locations using *P*-element-mediated transformation (67) (Figure 6A). In agreement with previous data (108), we found that, in the testes of adult males, the *stil* promoter directs reporter gene expression specifically in SpG and early SpCs (Figure 6B). Using RT-qPCR, we compared testes expression of one dose of *lacZ* reporter inserted in seven genomic sites on the X chromosome as well as in twelve sites on unpaired autosomes (i.e., when autosomes are opposed with balancer chromosomes (Supplementary Table S1)). Median expression of one copy of the reporter inserted in the X chromosome appears to be 1.8-fold higher than that in autosomes hemizygous for insertions (*P* = 0.005, M–W *U*-test; Figure 6C). When autosomal homologues were paired, the median expression of one copy of the reporter inserted in the autosomes was even lower than that in the unpaired state (median *X*/*A* ratio 2.4, *P* = 0.016, M–W *U*-test; Supplementary Figure S16). Therefore, the X chromosome indeed has the most favorable environment for transcription in SpG and in early SpCs.

We corroborated these results by analyzing chromatin accessibility to Dam-methylation (109,110) of the X chromosome and autosomes in SpG. Consistent with the transgene-based experiments, Dam-methylation, on average, appears to be 1.2-fold enhanced in the X chromosomal inter-LADs, compared to the pseudo-haploid dose of autosomal inter-LADs (Figure 6D). Moreover, positive values of the PC1, representing an active Hi-C compartment, are higher in the X chromosome than in autosomes in both SpG and SpCs (median X/A ratios 1.34 and 1.33, *P* = 10^–17^ and *P* = 10^–15^ in SpG and SpCs, respectively, M–W *U*-test; Figure 6E). Accordingly, active and inactive compartments in the X chromosome are stronger than that in autosomes at both stages (median X/A ratios for active compartment 1.14; Figure 6F), thus indicating that the active compartment is more spatially isolated from the inactive one in the X chromosome compared to autosomes. In line with these findings, contact frequency at mid- to long-range distances in the X chromosome is elevated relative to autosomes in both SpG and SpCs (*P* = 0.02, M–W *U*-test; Figure 6G). Finally, we found that the frequency of contacts between active genes positively correlates with their expression: the higher the expression, the stronger the interactions (Figure 6H). Therefore, active chromatin of the X chromosome in SpG is more ‘open’ and compartmentalized than that of autosomes.

## DISCUSSION

### Activation of a subset of SpC-specific genes correlates with the weakening of their promoter contacts with the NL

Consistent with previous studies in mammals and *Drosophila* (40,42), we found that almost all ubiquitously expressed promoters are localized in the inter-LADs (Figure 1D). We also found that promoters of testis-specific genes which are expressed in SpG and continue to be active in SpCs reside mainly in the inter-LADs in both SpG and SpCs (Figure 1E), thus supporting the idea of poor compatibility of gene expression with the localization at the NL. More importantly, we report here that initiation of transcription of the Aly-independent SpC-specific genes in the early SpCs (101) correlates with their detachment from the NL in this cell type (Figure 1F).

Surprisingly, promoters of Aly-dependent SpC-specific genes are mostly localized in LADs in SpCs (Figure 1C, G, Supplementary Figure S5). Since previously we have shown by FISH that testis-specific gene cluster from the *60D* region containing five Aly-dependent genes is mostly shifted from the NL to the nuclear interior in SpCs (102), there is an obvious contradiction between FISH and DamID data. It is reasonable to suggest that Aly-dependent genes start to express later than *aly*, whose expression is initiated in the early SpCs (99), i.e. in mid to mature SpCs. We then suppose that Aly-dependent SpC-specific genes may become hypermethylated by Dam-Lam in the early SpCs, where they stay in contact with the NL. In the absence of cell division, they may remain hypermethylated until the end of the mature SpC stage, even after losing the association with the NL in mature SpCs. This scenario implies that Aly-dependent SpC-specific genes lose their association with the NL in mature SpCs, where they become activated, but we could not observe weakening of their interactions with the NL due to technical limitations of the DamID procedure.

In summary, we conclude that, similarly to what was observed in mammals (40,45), the initiation of tissue-specific transcription in *Drosophila* correlates with the detachment of at least some types of promoters from the NL.

### Activation of SpC-specific genes correlates with their enhanced interactions and with partial topological insulation of the neighboring genome regions

**Figure 7.**
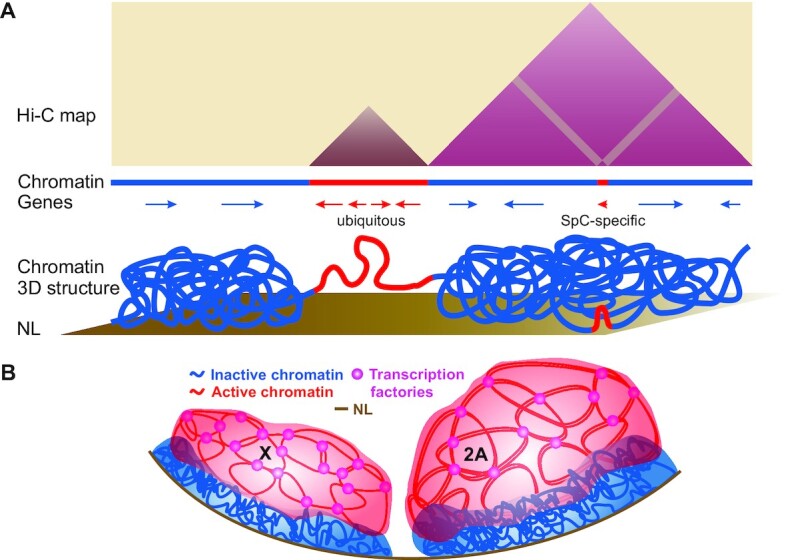
Working models explaining the obtained results. (**A**) A model demonstrating that initiation of SpC-specific transcription within inactive chromatin induces only local detachment of SpC-specific gene from the NL as well as only local insulation of the nearby regions, but not the segregation of inactive TAD into sub-TADs. Active and inactive chromatin is depicted in red and blue color, respectively. We propose that short region of active chromatin corresponding to the activated SpC-specific gene is not sufficient to split large inactive TAD globule into two parts. (**B**) A model of non-canonical dosage compensation mechanism that potentially operates in *Drosophila* male germline cells. We propose that in the male germline, single X chromosome adopts special 3D organization manifested in the enhanced interactions within its active compartment and in the stronger interactions of its LADs with the NL. We hypothesize that these enhanced interactions would lead to more frequent gathering of active X-linked genes into the transcription factories resulting in their up-regulation, and to more repressed state of low-expressed X-linked genes.

Previously, we and others have shown that TAD boundaries are enriched in housekeeping genes (3,18,31–33). Here, we report that initiation of SpC-specific transcription in SpCs correlates with partial spatial insulation of adjacent genome regions (Figure 3D, E). Since gene expression is accompanied by histone acetylation, our data support the hypothesis that *de novo* histone acetylation within inactive TADs can provoke spatial segregation of active and inactive chromatin (18). Yet, this rarely, if ever, leads to disintegration of these inactive TADs into smaller sub-TADs. The SpC-specific genes are rather small and more spaced in *Drosophila* genome than ubiquitous genes which are mainly organized in dense clusters (median distance between a pair of SpC-specific or ubiquitous genes is 58 kb versus 6.7 kb, respectively). Such short segments of active chromatin may be insufficient to notably perturb 3D organization of long regions of inactive chromatin within which they emerged. In support of this idea, our polymer modeling has shown that short active regions are looped out from the inactive TAD globule, whereas long active regions result in the partitioning of this globule into different sub-TADs (111) (Figure 7A). Additionally, architectural proteins which were shown to affect boundary strength (12,33,112) may be required to generate new boundaries. Thus, although new TAD boundaries do appear (Supplementary Fig. S8), their emergence may be associated with the enhancement of ubiquitous gene expression and/or with the binding of architectural proteins.

Our findings are in line with the accumulating data showing that genome architecture remains largely unaltered after the initiation of tissue-specific transcription in variety of organisms. For example, partitioning into TADs was not notably changed upon induction of transcriptional response by extracellular signals in mammalian cells (113–115). Furthermore, although activation of tissue-specific transcription within CTCF-independent TADs was shown to correlate with topological insulation during mouse neural differentiation, artificial recruitment of VP64 activator followed by transcriptional activation appears to be insufficient for creating TAD boundaries *de novo* (116). In *Drosophila* embryos, TAD demarcation was also revealed to be independent of tissue-specific transcription (33,117). Moreover, activation of transposons induced by depletion of Piwi in ovarian somatic cells correlates with transposon detachment from the NL and with increased spatial insulation of nearby regions, but does not cause the appearance of new TAD boundaries (118).

It is known that ∼40% of testis-specific genes are organized into clusters along the genome (102,119). This non-random linear genome arrangement may be necessary not only for the coordinated repression of testis-specific genes in somatic tissues, as was previously suggested (102,119), but also for the association of SpC-specific genes in the nuclear space upon activation. Indeed, one of the most striking findings of our study is that, upon activation, SpC-specific genes appear in close proximity with each other in the 3D nuclear space. Their interactions are more frequent than interactions between the inactive neighboring loci and more frequent than expected from the average contact probability decay (Figure 3G, J). Moreover, using 3C analysis, we found that spatial contacts between two SpC-specific genes from the *60D* region are notably increased in SpCs compared to SpG (Figure 3K). While it was demonstrated that housekeeping genes in *Drosophila* are clustered in the nuclear space (12,31,33) (Figure 3F), our findings point to *de novo* spatial clustering of tissue-specific genes upon activation. Given that increased contacts between active genes positively correlate with their expression level (12) (Figure 6H), as well as with Pol II and cohesin occupancy at these genes (16), their gene associations may create a favorable microenvironment for transcription. These findings update the hypothesis of transcription factories, according to which several genes simultaneously associate in the nuclear space to promote transcription and/or transcript processing (120, reviewed in (106)). Spatial clustering of active genes in the transcription factories enriched with Pol II was recently observed in wheat (121).

### Specific structural organization of the single X chromosome in *Drosophila* male germline cells mediates non-canonical dosage compensation

Several groups which have analyzed testes transcriptomes came to contradictory conclusions about the existence and magnitude of dosage compensation in *Drosophila* male germline cells (56,59–61). For example, a recent RNA-seq study performed on the isolated SpG and SpC cysts has not revealed ‘obvious dosage compensation of X-chromosomal genes’ in male germline cells (60). However, after subtracting the impact of somatic cyst cells from the data presented on Figure 6B of that paper, it becomes clear that partial dosage compensation ensuring ∼1.2–1.4-fold enhanced X-linked gene expression operates in SpG and SpCs. In the present work, we found strong evidence for the existence of partial dosage compensation in SpG and suggested mechanistic explanations of its non-canonical nature. Our transgene assay demonstrates that, in SpG and early SpCs, a *stil-lacZ* reporter is transcribed stronger when inserted in the X chromosome than when inserted in autosomes (Figure 6C). These results strongly support the existence of X chromosome-based transcriptional activation in early germline cells of *Drosophila* testes. However, the magnitude of this activation may be lower than 1.8-fold, since the number of transgene insertions analyzed was not very large.

The male X chromosome has no homologue, which clearly distinguishes it from autosomes. In this study, we demonstrate that, in both SpG and SpCs, the single X chromosome interacts stronger with the NL and is positioned closer to it than autosomes (Figure 4). In agreement with the idea suggested by Kind *et al.* (122), we propose that LADs compete for NL binding because there is a limited surface area for these interactions. While each pair of autosomal homologues occupy CT, which is roughly double the volume of a single X chromosome (Supplementary Figure S12), the surface of the NL, opposing CTs, may not be proportionally enlarged. This volume-to-surface ratio may explain the greater proportion of LADs bound to the NL in the single X chromosome compared to autosomes. The idea of competition between LADs for NL binding is consistent with the fact that, in mammals, only <30% of all LADs revealed in cell population are bound to the NL in individual cells. In addition, haploid chromosomes have stronger interactions with the NL than diploid chromosomes (122,123).

We further propose that stronger interactions of the male X chromosome with the NL lead to more prominent spatial segregation of its active and inactive compartments (Figure 6F), resulting in a more pronounced active compartment in the X chromosome compared to autosomes (Figure 6E). This idea is supported by our previous results showing that the active compartment is weakened upon depletion of Lam Dm0 in *Drosophila* S2 cells, followed by the detachment of LADs from the NL (37). We note that the influence of CT-NL attachment on the formation of the active compartment is rather mild, at least in S2 cells, since interactions within the active autosomal compartment became only ∼3% weaker upon NL disruption.

The absence of a homologue may affect the active compartment in another way, which was recently suggested by Pal *et al.* (93) through the use of polymer modeling. This group showed that the absence of a paired homologue makes the single X chromosome more flexible and prone to increased mid- and long-range contacts. In support of this idea, our transgene experiments demonstrate that expression of a hemizygous reporter inserted in the paired autosomes is weaker than in the unpaired autosomes (Figure 6C and Supplementary Figure S16). Therefore, both the increased binding of LADs with the NL and more flexible state of the single X chromosome may specifically shape its 3D organization leading to more frequent gathering of active promoters into transcription factories and to the enhanced transcription of the X-linked genes (Figure 7B). The latter idea is based on the observed positive correlation between gene-to-gene contact frequency within active compartment and gene expression (12) (Figure 6H). A decline in the expression of actively expressed genes (including SpC-specific genes) upon Lam-KD in SpCs (Figure 5C, D) also supports the role of the NL in the maintenance of normal gene expression. Furthermore, spatial clustering of active gene promoters in *Drosophila* was shown to facilitate their transcription (124). Yet, stronger interactions of the single X chromosome with the NL do not lead to the preferential down-regulation of its active genes upon Lam-KD in SpCs (Figure 5C). However, this may be explained by an existence of other mechanisms that affect X-linked gene expression in SpCs, compared to SpG, such as the reduced phosphorylation of Pol II C-terminal domain specifically on the X chromosome (61). On the other hand, stronger association of the single X chromosome with the NL results in a more prominent repression of its weakly expressed genes (Figure 5C). The enhanced repression is likely necessary to counteract the spreading of active chromatin on these genes. Our results do not support the hypothesis that ‘the unpaired X chromosomes of males have weaker repressive domains than the same domains in the paired X chromosomes of females’ (125).

Our mechanistic explanation of non-canonical dosage compensation may be applicable not only for male germline cells but for male somatic cells as well. The ‘hard-wired architecture’ (126) of the X chromosome in male somatic cells may be not only the prerequisite for spreading of the MSL complex on genes for their ∼1.4-fold activation by the canonical dosage compensation mechanism, as was suggested by Ramírez *et al.* (127). Such 3D organization may also ensure an additional ∼1.4-fold up-regulation of these genes by a non-canonical mechanism, resulting in two-fold up-regulation in total. In the absence of the MSL complex, the specific 3D organization of the X chromosome in male germline cells leads to only ∼1.2–1.4-fold up-regulation of X-linked genes by a non-canonical mechanism.

In conclusion, we found that, at least for some tissue-specific genes, transcription initiation correlates with the detachment of their promoters from the NL, with topological insulation of adjoining regions, and with more frequent association of their promoters with each other in the nucleus space. We also found that, in the male germline cells, the single X chromosome occupies a more proximal position and stronger interacts with the NL than autosomes, which correlates with stronger repression of weakly expressed X-linked genes. At the same time, the X chromosome contains a more ‘open’ and self-interacting active compartment than autosomes. The latter features of X chromosome organization correspond to preferential up-regulation of X-linked transgenes. We propose that a non-canonical mechanism of partial dosage compensation operates in male germline cells. It is based on the intrinsic ability of the single X chromosome to adopt more compartmentalized and more flexible 3D conformation, which results in higher expression of X-linked active genes, as well as in stronger repression of X-linked inactive genes.

## DATA AVAILABILITY

All raw and processed Lam-DamID-seq, RNA-seq and Hi-C data were deposited in the NCBI Gene Expression Omnibus (GEO) under the accession numbers GSE157565 (for Lam-DamID-seq and RNA-seq) and GSE156567 (for Hi-C). In-house scripts are available at GitHub (https://github.com/foriin/Lam_Sperm). The link for UCSC Browser (https://genome.ucsc.edu/s/Shevelyov/LaminDamID%20in%20SpG%20and%20SpCs).

## Supplementary Material

gkac109_Supplemental_Files

## References

[1] Lieberman-Aiden E. , van Berkum N.L., Williams L., Imakaev M., Ragoczy T., Telling A., Amit I., Lajoie B.R., Sabo P.J., Dorschner M.O. et al. Comprehensive mapping of long-range interactions reveals folding principles of the human genome. Science. 2009; 326:289–293. 19815776 10.1126/science.1181369 PMC2858594

[2] Dixon J.R. , Selvaraj S., Yue F., Kim A., Li Y., Shen Y., Hu M., Liu J.S., Ren B. Topological domains in mammalian genomes identified by analysis of chromatin interactions. Nature. 2012; 485:376–380. 22495300 10.1038/nature11082 PMC3356448

[3] Hou C. , Li L., Qin Z.S., Corces V.G. Gene density, transcription, and insulators contribute to the partition of the *Drosophila* genome into physical domains. Mol. Cell. 2012; 48:471–484. 23041285 10.1016/j.molcel.2012.08.031 PMC3496039

[4] Nora E.P. , Lajoie B.R., Schulz E.G., Giorgetti L., Okamoto I., Servant N., Piolot T., van Berkum N.L., Meisig J., Sedat J. et al. Spatial partitioning of the regulatory landscape of the X-inactivation centre. Nature. 2012; 485:381–385. 22495304 10.1038/nature11049 PMC3555144

[5] Sexton T. , Yaffe E., Kenigsberg E., Bantignies F., Leblanc B., Hoichman M., Parrinello H., Tanay A., Cavalli G. Three-dimensional folding and functional organization principles of the *Drosophila* genome. Cell. 2012; 148:458–472. 22265598 10.1016/j.cell.2012.01.010

[6] Flyamer I.M. , Gassler J., Imakaev M., Brandão H.B., Ulianov S.V., Abdennur N., Razin S.V., Mirny L.A., Tachibana-Konwalski K. Single-nucleus Hi-C reveals unique chromatin reorganization at oocyte-to-zygote transition. Nature. 2017; 544:110–114. 28355183 10.1038/nature21711 PMC5639698

[7] Bintu B. , Mateo L.J., Su J.H., Sinnott-Armstrong N.A., Parker M., Kinrot S., Yamaya K., Boettiger A.N., Zhuang X. Super-resolution chromatin tracing reveals domains and cooperative interactions in single cells. Science. 2018; 362:eaau1783. 30361340 10.1126/science.aau1783 PMC6535145

[8] Szabo Q. , Jost D., Chang J.M., Cattoni D.I., Papadopoulos G.L., Bonev B., Sexton T., Gurgo J., Jacquier C., Nollmann M. et al. TADs are 3D structural units of higher-order chromosome organization in *Drosophil**a*. Sci. Adv. 2018; 4:eaar8082. 29503869 10.1126/sciadv.aar8082 PMC5829972

[9] Ulianov S.V. , Zakharova V.V., Galitsyna A.A., Kos P.I., Polovnikov K.E., Flyamer I.M., Mikhaleva E.A., Khrameeva E.E., Germini D., Logacheva M.D. et al. Order and stochasticity in the folding of individual *Drosophila* genomes. Nat. Commun. 2021; 12:41. 33397980 10.1038/s41467-020-20292-z PMC7782554

[10] Rao S.S. , Huntley M.H., Durand N.C., Stamenova E.K., Bochkov I.D., Robinson J.T., Sanborn A.L., Machol I., Omer A.D., Lander E.S. et al. A 3D map of the human genome at kilobase resolution reveals principles of chromatin looping. Cell. 2014; 159:1665–1680. 25497547 10.1016/j.cell.2014.11.021 PMC5635824

[11] Eagen K.P. , Aiden E.L., Kornberg R.D. Polycomb-mediated chromatin loops revealed by a subkilobase-resolution chromatin interaction map. Proc. Natl. Acad. Sci. U.S.A. 2017; 114:8764–8769. 28765367 10.1073/pnas.1701291114 PMC5565414

[12] Rowley M.J. , Nichols M.H., Lyu X., Ando-Kuri M., Rivera I.S.M., Hermetz K., Wang P., Ruan Y., Corces V.G Evolutionarily conserved principles predict 3D chromatin organization. Mol. Cell. 2017; 67:837–852. 28826674 10.1016/j.molcel.2017.07.022 PMC5591081

[13] Stadler M.R. , Haines J.E., Eisen M.B. Convergence of topological domain boundaries, insulators, and polytene interbands revealed by high-resolution mapping of chromatin contacts in the early *Drosophila**melanogaster* embryo. Elife. 2017; 6:e29550. 29148971 10.7554/eLife.29550 PMC5739541

[14] Wang Q. , Sun Q., Czajkowsky D.M., Shao Z. Sub-kb Hi-C in *D. melanogaster* reveals conserved characteristics of TADs between insect and mammalian cells. Nat. Commun. 2018; 9:188. 29335463 10.1038/s41467-017-02526-9 PMC5768742

[15] Chathoth K.T. , Zabet N.R. Chromatin architecture reorganization during neuronal cell differentiation in *Drosophila* genome. Genome Res. 2019; 29:613–625. 30709849 10.1101/gr.246710.118 PMC6442379

[16] Rowley M.J. , Lyu X., Rana V., Ando-Kuri M., Karns R., Bosco G., Corces V.G. Condensin II counteracts cohesin and RNA polymerase II in the establishment of 3D chromatin organization. Cell Rep. 2019; 26:2890–2903. 30865881 10.1016/j.celrep.2019.01.116 PMC6424357

[17] Krietenstein N. , Abraham S., Venev S.V., Abdennur N., Gibcus J., Hsieh T.S., Parsi K.M., Yang L., Maehr R., Mirny L.A. et al. Ultrastructural details of mammalian chromosome architecture. Mol. Cell. 2020; 78:554–565. 32213324 10.1016/j.molcel.2020.03.003 PMC7222625

[18] Ulianov S.V. , Khrameeva E.E., Gavrilov A.A., Flyamer I.M., Kos P., Mikhaleva E.A., Penin A.A., Logacheva M.D., Imakaev M.V., Chertovich A. et al. Active chromatin and transcription play a key role in chromosome partitioning into topologically associating domains. Genome Res. 2016; 26:70–84. 26518482 10.1101/gr.196006.115 PMC4691752

[19] Sanborn A.L. , Rao S.S., Huang S.C., Durand N.C., Huntley M.H., Jewett A.I., Bochkov I.D., Chinnappan D., Cutkosky A., Li J. et al. Chromatin extrusion explains key features of loop and domain formation in wild-type and engineered genomes. Proc. Natl. Acad. Sci. U.S.A. 2015; 112:E6456–E6465. 26499245 10.1073/pnas.1518552112 PMC4664323

[20] Fudenberg G. , Imakaev M., Lu C., Goloborodko A., Abdennur N., Mirny L.A. Formation of chromosomal domains by loop extrusion. Cell Rep. 2016; 15:2038–2049. 27210764 10.1016/j.celrep.2016.04.085 PMC4889513

[21] Rao S.S.P. , Huang S.C., St Hilaire G.B., Engreitz J.M., Perez E.M., Kieffer-Kwon K.R., Sanborn A.L., Johnstone S.E., Bascom G.D., Bochkov I.D. et al. Cohesin loss eliminates all loop domains. Cell. 2007; 171:305–320. 10.1016/j.cell.2017.09.026 PMC5846482 28985562

[22] Schwarzer W. , Abdennur N., Goloborodko A., Pekowska A., Fudenberg G., Loe-Mie Y., Fonseca N.A., Huber W., Haering C.H., Mirny L. et al. Two independent modes of chromatin organization revealed by cohesin removal. Nature. 2017; 551:51–56. 29094699 10.1038/nature24281 PMC5687303

[23] Kaushal A. , Mohana G., Dorier J., Özdemir I., Omer A., Cousin P., Semenova A., Taschner M., Dergai O., Marzetta F. et al. CTCF loss has limited effects on global genome architecture in *Drosophila* despite critical regulatory functions. Nat. Commun. 2021; 12:1011. 33579945 10.1038/s41467-021-21366-2 PMC7880997

[24] Falk M. , Feodorova Y., Naumova N., Imakaev M., Lajoie B.R., Leonhardt H., Joffe B., Dekker J., Fudenberg G., Solovei I. et al. Heterochromatin drives compartmentalization of inverted and conventional nuclei. Nature. 2019; 570:395–399. 31168090 10.1038/s41586-019-1275-3 PMC7206897

[25] Wang L. , Gao Y., Zheng X., Liu C., Dong S., Li R., Zhang G., Wei Y., Qu H., Li Y. et al. Histone modifications regulate chromatin compartmentalization by contributing to a phase separation mechanism. Mol. Cell. 2019; 76:646–659. 31543422 10.1016/j.molcel.2019.08.019

[26] Lee Y.C.G. , Ogiyama Y., Martins N.M.C., Beliveau B.J., Acevedo D., Wu C.T., Cavalli G., Karpen G.H. Pericentromeric heterochromatin is hierarchically organized and spatially contacts H3K9me2 islands in euchromatin. PLoS Genet. 2020; 16:e1008673. 32203508 10.1371/journal.pgen.1008673 PMC7147806

[27] Razin S.V. , Gavrilov A.A. The role of liquid–liquid phase separation in the compartmentalization of cell nucleus and spatial genome organization. Biochemistry (Mosc.). 2020; 85:643–650. 32586227 10.1134/S0006297920060012

[28] Larson A.G. , Elnatan D., Keenen M.M., Trnka M.J., Johnston J.B., Burlingame A.L., Agard D.A., Redding S., Narlikar G.J. Liquid droplet formation by HP1α suggests a role for phase separation in heterochromatin. Nature. 2017; 547:236–240. 28636604 10.1038/nature22822 PMC5606208

[29] Strom A.R. , Emelyanov A.V., Mir M., Fyodorov D.V., Darzacq X., Karpen G.H. Phase separation drives heterochromatin domain formation. Nature. 2017; 547:241–245. 28636597 10.1038/nature22989 PMC6022742

[30] Gibson B.A. , Doolittle L.K., Schneider M.W.G., Jensen L.E., Gamarra N., Henry L., Gerlich D.W., Redding S., Rosen M.K. Organization of chromatin by intrinsic and regulated phase separation. Cell. 2019; 179:470–484. 31543265 10.1016/j.cell.2019.08.037 PMC6778041

[31] Cubeñas-Potts C. , Rowley M.J., Lyu X., Li G., Lei E.P., Corces V.G. Different enhancer classes in *Drosophila* bind distinct architectural proteins and mediate unique chromatin interactions and 3D architecture. Nucleic Acids Res. 2017; 45:1714–1730. 27899590 10.1093/nar/gkw1114 PMC5389536

[32] El-Sharnouby S. , Fischer B., Magbanua J.P., Umans B., Flower R., Choo S.W., Russell S., White R. Regions of very low H3K27me3 partition the *Drosophila* genome into topological domains. PLoS One. 2017; 12:e0172725. 28282436 10.1371/journal.pone.0172725 PMC5345799

[33] Hug C.B. , Grimaldi A.G., Kruse K., Vaquerizas J.M. Chromatin architecture emerges during zygotic genome activation independent of transcription. Cell. 2017; 169:216–228. 28388407 10.1016/j.cell.2017.03.024

[34] Gruenbaum Y. , Foisner R. Lamins: nuclear intermediate filament proteins with fundamental functions in nuclear mechanics and genome regulation. Annu. Rev. Biochem. 2015; 84:131–164. 25747401 10.1146/annurev-biochem-060614-034115

[35] Shevelyov Y.Y. , Ulianov S.V. The nuclear lamina as an organizer of chromosome architecture. Cells. 2019; 8:2. 10.3390/cells8020136 PMC6406483 30744037

[36] Zheng X. , Hu J., Yue S., Kristiani L., Kim M., Sauria M., Taylor J., Kim Y., Zheng Y. Lamins organize the global three-dimensional genome from the nuclear periphery. Mol. Cell. 2018; 71:802–815. 30201095 10.1016/j.molcel.2018.05.017 PMC6886264

[37] Ulianov S.V. , Doronin S.A., Khrameeva E.E., Kos P.I., Luzhin A.V., Starikov S.S., Galitsyna A.A., Nenasheva V.V., Ilyin A.A., Flyamer I.M. et al. Nuclear lamina integrity is required for proper spatial organization of chromatin in *Drosophil**a*. Nat. Commun. 2019; 10:1176. 30862957 10.1038/s41467-019-09185-y PMC6414625

[38] Guelen L. , Pagie L., Brasset E., Meuleman W., Faza M.B., Talhout W., Eussen B.H., de Klein A., Wessels L., de Laat W. et al. Domain organization of human chromosomes revealed by mapping of nuclear lamina interactions. Nature. 2008; 453:948–951. 18463634 10.1038/nature06947

[39] Ikegami K. , Egelhofer T.A., Strome S., Lieb J.D. *Caenorhabditis elegans* chromosome arms are anchored to the nuclear membrane via discontinuous association with LEM-2. Genome Biol. 2010; 11:R120. 21176223 10.1186/gb-2010-11-12-r120 PMC3046480

[40] Peric-Hupkes D. , Meuleman W., Pagie L., Bruggeman S.W., Solovei I., Brugman W., Gräf S., Flicek P., Kerkhoven R.M., van Lohuizen M. et al. Molecular maps of the reorganization of genome-nuclear lamina interactions during differentiation. Mol. Cell. 2010; 38:603–613. 20513434 10.1016/j.molcel.2010.03.016 PMC5975946

[41] van Bemmel J.G. , Pagie L., Braunschweig U., Brugman W., Meuleman W., Kerkhoven R.M., van Steensel B. The insulator protein SU(HW) fine-tunes nuclear lamina interactions of the *Drosophila* genome. PLoS One. 2010; 5:e15013. 21124834 10.1371/journal.pone.0015013 PMC2991331

[42] Pindyurin A.V. , Ilyin A.A., Ivankin A.V., Tselebrovsky M.V., Nenasheva V.V., Mikhaleva E.A., Pagie L., van Steensel B., Shevelyov Y.Y. The large fraction of heterochromatin in *Drosophila* neurons is bound by both B-type lamin and HP1a. Epigenetics Chromatin. 2018; 11:65. 30384843 10.1186/s13072-018-0235-8 PMC6211408

[43] Wu F. , Yao J. Spatial compartmentalization at the nuclear periphery characterized by genome-wide mapping. BMC Genomics. 2013; 14:591. 23987233 10.1186/1471-2164-14-591 PMC3849850

[44] Wu F. , Yao J. Identifying novel transcriptional and epigenetic features of nuclear lamina-associated genes. Sci. Rep. 2017; 7:100. 28273906 10.1038/s41598-017-00176-x PMC5427898

[45] Brueckner L. , Zhao P.A., van Schaik T., Leemans C., Sima J., Peric-Hupkes D., Gilbert D.M., van Steensel B. Local rewiring of genome-nuclear lamina interactions by transcription. EMBO J. 2020; 39:e103159. 32080885 10.15252/embj.2019103159 PMC7073462

[46] Shevelyov Y.Y. , Nurminsky D.I. The nuclear lamina as a gene-silencing hub. Curr. Issues Mol. Biol. 2012; 14:27–38. 21795760

[47] Fuller M.T. Genetic control of cell proliferation and differentiation in *Drosophila* spermatogenesis. Semin. Cell Dev. Biol. 1998; 9:433–444. 9813190 10.1006/scdb.1998.0227

[48] Hiller M. , Chen X., Pringle M.J., Suchorolski M., Sancak Y., Viswanathan S., Bolival B., Lin T.Y., Marino S., Fuller M.T. Testis-specific TAF homologs collaborate to control a tissue-specific transcription program. Development. 2004; 131:5297–5308. 15456720 10.1242/dev.01314

[49] Beall E.L. , Lewis P.W., Bell M., Rocha M., Jones D.L., Botchan M.R. Discovery of tMAC: a *Drosophila* testis-specific meiotic arrest complex paralogous to myb-muvB. Genes Dev. 2007; 21:904–919. 17403774 10.1101/gad.1516607 PMC1847709

[50] White-Cooper H. , Caporilli S. Transcriptional and post-transcriptional regulation of *Drosophila* germline stem cells and their differentiating progeny. Adv. Exp. Med. Biol. 2013; 786:47–61. 23696351 10.1007/978-94-007-6621-1_4

[51] Hilfiker A. , Hilfiker-Kleiner D., Pannuti A., Lucchesi J.C. *mof*, a putative acetyl transferase gene related to the tip60 and MOZ human genes and to the SAS genes of yeast, is required for dosage compensation in *Drosophil**a*. EMBO J. 1997; 16:2054–2060. 9155031 10.1093/emboj/16.8.2054 PMC1169808

[52] Akhtar A. , Becker P.B. Activation of transcription through histone H4 acetylation by MOF, an acetyltransferase essential for dosage compensation in *Drosophil**a*. Mol. Cell. 2000; 5:367–375. 10882077 10.1016/s1097-2765(00)80431-1

[53] Smith E.R. , Pannuti A., Gu W., Steurnagel A., Cook R.G., Allis C.D., Lucchesi J.C. The *Drosophila* MSL complex acetylates histone H4 at lysine 16, a chromatin modification linked to dosage compensation. Mol. Cell. Biol. 2000; 20:312–318. 10594033 10.1128/mcb.20.1.312-318.2000 PMC85086

[54] Kuroda M.I. , Hilfiker A., Lucchesi J.C. Dosage compensation in *Drosophila* – a model for the coordinate regulation of transcription. Genetics. 2016; 204:435–450. 27729494 10.1534/genetics.115.185108 PMC5068838

[55] Zhang Y. , Malone J.H., Powell S.K., Periwal V., Spana E., Macalpine D.M., Oliver B. Expression in aneuploid *Drosophila* S2 cells. PLoS Biol. 2010; 8:e1000320. 20186269 10.1371/journal.pbio.1000320 PMC2826376

[56] Meiklejohn C.D. , Landeen E.L., Cook J.M., Kingan S.B., Presgraves D.C. Sex chromosome-specific regulation in the *Drosophila* male germline but little evidence for chromosomal dosage compensation or meiotic inactivation. PLoS Biol. 2011; 9:e1001126. 21857805 10.1371/journal.pbio.1001126 PMC3156688

[57] Lott S.E. , Villalta J.E., Schroth G.P., Luo S., Tonkin L.A., Eisen M.B. Noncanonical compensation of zygotic X transcription in early *Drosophila**melanogaster* development revealed through single-embryo RNA-seq. PLoS Biol. 2011; 9:e1000590. 21346796 10.1371/journal.pbio.1000590 PMC3035605

[58] Rastelli L. , Kuroda M.I. An analysis of maleless and histone H4 acetylation in *Drosophila**melanogaster* spermatogenesis. Mech. Dev. 1998; 71:107–117. 9507080 10.1016/s0925-4773(98)00009-4

[59] Deng X. , Hiatt J.B., Nguyen D.K., Ercan S., Sturgill D., Hillier L.W., Schlesinger F., Davis C.A., Reinke V.J., Gingeras T.R. et al. Evidence for compensatory upregulation of expressed X-linked genes in mammals, *Caenorhabditis**elegans* and *Drosophila**melanogaster*. Nat. Genet. 2011; 43:1179–1185. 22019781 10.1038/ng.948 PMC3576853

[60] Shi Z. , Lim C., Tran V., Cui K., Zhao K., Chen X. Single-cyst transcriptome analysis of *Drosophila* male germline stem cell lineage. Development. 2020; 147:dev184259. 32122991 10.1242/dev.184259 PMC7174844

[61] Mahadevaraju S. , Fear J.M., Akeju M., Galletta B.J., Pinheiro M.M.L.S., Avelino C.C., Cabral-de-Mello D.C., Conlon K., Dell’Orso S., Demere Z. et al. Dynamic sex chromosome expression in *Drosophila* male germ cells. Nat. Commun. 2021; 12:892. 33563972 10.1038/s41467-021-20897-y PMC7873209

[62] Markstein M. , Pitsouli C., Villalta C., Celniker S.E., Perrimon N. Exploiting position effects and the gypsy retrovirus insulator to engineer precisely expressed transgenes. Nat. Genet. 2008; 40:476–483. 18311141 10.1038/ng.101 PMC2330261

[63] Bischof J. , Maeda R.K., Hediger M., Karch F., Basler K. An optimized transgenesis system for *Drosophila* using germ-line-specific phiC31 integrases. Proc. Natl. Acad. Sci. U.S.A. 2007; 104:3312–3317. 17360644 10.1073/pnas.0611511104 PMC1805588

[64] Laktionov P.P. , White-Cooper H., Maksimov D.A., Belyakin S.N. Transcription factor COMR acts as a direct activator in the genetic program controlling spermatogenesis in *D. melanogaster*. Mol. Biol. (Mosk). 2014; 48:130–140. 25842836

[65] McKearin D.M. , Spradling A.C. *Bag-of-marbles*: a *Drosophila* gene required to initiate both male and female gametogenesis. Genes Dev. 1990; 4:2242–2251. 2279698 10.1101/gad.4.12b.2242

[66] Chen D. , McKearin D.M. A discrete transcriptional silencer in the *bam* gene determines asymmetric division of the *Drosophila**germline* stem cell. Development. 2003; 130:1159–1170. 12571107 10.1242/dev.00325

[67] Rubin G.M. , Spradling A.C. Genetic transformation of *Drosophila* with transposable element vectors. Science. 1982; 218:348–353. 6289436 10.1126/science.6289436

[68] Pickersgill H. , Kalverda B., de Wit E., Talhout W., Fornerod M., van Steensel B. Characterization of the *Drosophila**melanogaster* genome at the nuclear lamina. Nat. Genet. 2006; 38:1005–1014. 16878134 10.1038/ng1852

[69] Southall T.D. , Gold K.S., Egger B., Davidson C.M., Caygill E.E., Marshall O.J., Brand A.H. Cell-type-specific profiling of gene expression and chromatin binding without cell isolation: assaying RNA pol II occupancy in neural stem cells. Dev. Cell. 2013; 26:101–112. 23792147 10.1016/j.devcel.2013.05.020 PMC3714590

[70] Thummel C.S. , Boulet A.M., Lipshitz H.D. Vectors for *Drosophila* P-element-mediated transformation and tissue culture transfection. Gene. 1988; 74:445–456. 3246353 10.1016/0378-1119(88)90177-1

[71] Gan Q. , Chepelev I., Wei G., Tarayrah L., Cui K., Zhao K., Chen X. Dynamic regulation of alternative splicing and chromatin structure in *Drosophila* gonads revealed by RNA-seq. Cell Res. 2010; 20:763–783. 20440302 10.1038/cr.2010.64 PMC2919574

[72] Chen X. , Lu C., Morillo Prado J.R., Eun S.H., Fuller M.T. Sequential changes at differentiation gene promoters as they become active in a stem cell lineage. Development. 2011; 138:2441–2450. 21610025 10.1242/dev.056572 PMC3100706

[73] Kim J. , Lu C., Srinivasan S., Awe S., Brehm A., Fuller M.T. Blocking promiscuous activation at cryptic promoters directs cell type-specific gene expression. Science. 2017; 356:717–721. 28522526 10.1126/science.aal3096 PMC5572561

[74] Gönczy P. , Matunis E., DiNardo S. *Bag-of-marbles* and *benign gonial cell neoplasm* act in the germline to restrict proliferation during *Drosophila* spermatogenesis. Development. 1997; 124:4361–4371. 9334284 10.1242/dev.124.21.4361

[75] White-Cooper H. Tissue, cell type and stage-specific ectopic gene expression and RNAi induction in the *Drosophila* testis. Spermatogenesis. 2012; 2:11–22. 22553486 10.4161/spmg.19088 PMC3341242

[76] Thomas S.E. , McKee B.D. Analysis of chromosome dynamics and chromosomal proteins in *Drosophila* spermatocytes. Methods Mol. Biol. 2009; 558:217–234. 19685327 10.1007/978-1-60761-103-5_13

[77] Ilyin A.A. , Ryazansky S.S., Doronin S.A., Olenkina O.M., Mikhaleva E.A., Yakushev E.Y., Abramov Y.A., Belyakin S.N., Ivankin A.V., Pindyurin A.V. et al. Piwi interacts with chromatin at nuclear pores and promiscuously binds nuclear transcripts in *Drosophila* ovarian somatic cells. Nucleic Acids Res. 2017; 45:7666–7680. 28472469 10.1093/nar/gkx355 PMC5570135

[78] Gavrilov A.A. , Razin S.V. Spatial configuration of the chicken alpha-globin gene domain: immature and active chromatin hubs. Nucleic Acids Res. 2008; 36:4629–4640. 18621783 10.1093/nar/gkn429 PMC2504291

[79] Belyaeva E.S. , Zhimulev I.F., Volkova E.I., Alekseyenko A.A., Moshkin Y.M., Koryakov D.E. *Su(UR)ES*: a gene suppressing DNA underreplication in intercalary and pericentric heterochromatin of *Drosophila**melanogaster* polytene chromosomes. Proc. Natl. Acad. Sci. U.S.A. 1998; 95:7532–7537. 9636184 10.1073/pnas.95.13.7532 PMC22673

[80] Moshkin Y.M. , Belyakin S.N., Rubtsov N.B., Kokoza E.B., Alekseyenko A.A., Volkova E.I., Belyaeva E.S., Makunin I.V., Spierer P., Zhimulev I.F. Microdissection and sequence analysis of pericentric heterochromatin from the *Drosophila**melanogaster* mutant *suppressor**of**under replicatio**n*. Chromosoma. 2002; 111:114–125. 12111334 10.1007/s00412-002-0190-8

[81] Cremer M. , Grasser F., Lanctôt C., Müller S., Neusser M., Zinner R., Solovei I., Cremer T. Multicolor 3D fluorescence in situ hybridization for imaging interphase chromosomes. Methods Mol. Biol. 2008; 463:205–239. 18951171 10.1007/978-1-59745-406-3_15

[82] Stuurman N. , Maus N., Fisher P.A. Interphase phosphorylation of the *Drosophila* nuclear lamin: site-mapping using a monoclonal antibody. J. Cell Sci. 1995; 108:3137–3144. 8537453 10.1242/jcs.108.9.3137

[83] Riemer D. , Stuurman N., Berrios M., Hunter C., Fisher P.A., Weber K. Expression of *Drosophila* lamin c is developmentally regulated: analogies with vertebrate A-type lamins. J. Cell Sci. 1995; 108:3189–3198. 7593280 10.1242/jcs.108.10.3189

[84] Simon J.A. , Sutton C.A., Lobell R.B., Glaser R.L., Lis J.T. Determinants of heat shock-induced chromosome puffing. Cell. 1985; 40:805–817. 3986904 10.1016/0092-8674(85)90340-x

[85] Chintapalli V.R. , Wang J., Dow J.A. Using flyatlas to identify better *Drosophila**melanogaster* models of human disease. Nat. Genet. 2007; 39:715–720. 17534367 10.1038/ng2049

[86] Laktionov P.P. , Maksimov D.A., Romanov S.E., Antoshina P.A., Posukh O.V., White-Cooper H., Koryakov D.E., Belyakin S.N. Genome-wide analysis of gene regulation mechanisms during *Drosophila* spermatogenesis. Epigenetics Chromatin. 2018; 11:14. 29609617 10.1186/s13072-018-0183-3 PMC5879934

[87] Langmead B. , Salzberg S.L. Fast gapped-read alignment with bowtie 2. Nat. Methods. 2012; 9:357–359. 22388286 10.1038/nmeth.1923 PMC3322381

[88] Anders S. , Pyl P.T., Huber W. HTSeq–a python framework to work with high-throughput sequencing data. Bioinformatics. 2015; 31:166–169. 25260700 10.1093/bioinformatics/btu638 PMC4287950

[89] Imakaev M. , Fudenberg G., McCord R.P., Naumova N., Goloborodko A., Lajoie B.R., Dekker J., Mirny L.A. Iterative correction of Hi-C data reveals hallmarks of chromosome organization. Nat. Methods. 2012; 9:999–1003. 22941365 10.1038/nmeth.2148 PMC3816492

[90] Yang T. , Zhang F., Yardımcı G.G., Song F., Hardison R.C., Noble W.S., Yue F., Li Q. HiCRep: assessing the reproducibility of Hi-C data using a stratum-adjusted correlation coefficient. Genome Res. 2017; 27:1939–1949. 28855260 10.1101/gr.220640.117 PMC5668950

[91] Filippova D. , Patro R., Duggal G., Kingsford C. Identification of alternative topological domains in chromatin. Algorithms Mol. Biol. 2014; 9:14. 24868242 10.1186/1748-7188-9-14 PMC4019371

[92] Crane E. , Bian Q., McCord R.P., Lajoie B.R., Wheeler B.S., Ralston E.J., Uzawa S., Dekker J., Meyer B.J. Condensin-driven remodelling of X chromosome topology during dosage compensation. Nature. 2015; 523:240–244. 26030525 10.1038/nature14450 PMC4498965

[93] Pal K. , Forcato M., Jost D., Sexton T., Vaillant C., Salviato E., Mazza E.M.C., Lugli E., Cavalli G., Ferrari F. Global chromatin conformation differences in the *Drosophila* dosage compensated chromosome X. Nat. Commun. 2019; 10:5355. 31767860 10.1038/s41467-019-13350-8 PMC6877619

[94] Patro R. , Duggal G., Love M.I., Irizarry R.A., Kingsford C. Salmon provides fast and bias-aware quantification of transcript expression. Nat. Methods. 2017; 14:417–419. 28263959 10.1038/nmeth.4197 PMC5600148

[95] Love M.I. , Huber W., Anders S. Moderated estimation of fold change and dispersion for RNA-seq data with DESeq2. Genome Biol. 2014; 15:550. 25516281 10.1186/s13059-014-0550-8 PMC4302049

[96] van Steensel B. , Henikoff S. Identification of in vivo DNA targets of chromatin proteins using tethered dam methyltransferase. Nat. Biotechnol. 2000; 18:424–428. 10748524 10.1038/74487

[97] Pindyurin A.V. , Pagie L., Kozhevnikova E.N., van Arensbergen J., van Steensel B. Inducible DamID systems for genomic mapping of chromatin proteins in *Drosophil**a*. Nucleic Acids Res. 2016; 44:5646–5657. 27001518 10.1093/nar/gkw176 PMC4937306

[98] Pindyurin A.V. Genome-wide cell type-specific mapping of in vivo chromatin protein binding using an FLP-inducible DamID system in *Drosophil**a*. Methods Mol. Biol. 2017; 1654:99–124. 28986785 10.1007/978-1-4939-7231-9_7

[99] White-Cooper H. , Leroy D., MacQueen A., Fuller M.T. Transcription of meiotic cell cycle and terminal differentiation genes depends on a conserved chromatin associated protein, whose nuclear localisation is regulated. Development. 2000; 127:5463–5473. 11076766 10.1242/dev.127.24.5463

[100] White-Cooper H. , Schäfer M.A., Alphey L.S., Fuller M.T. Transcriptional and post-transcriptional control mechanisms coordinate the onset of spermatid differentiation with meiosis i in *Drosophil**a*. Development. 1998; 125:125–134. 9389670 10.1242/dev.125.1.125

[101] Lu D. , Sin H.S., Lu C., Fuller M.T. Developmental regulation of cell type-specific transcription by novel promoter-proximal sequence elements. Genes Dev. 2020; 34:663–677. 32217666 10.1101/gad.335331.119 PMC7197356

[102] Shevelyov Y.Y. , Lavrov S.A., Mikhaylova L.M., Nurminsky I.D., Kulathinal R.J., Egorova K.S., Rozovsky Y.M., Nurminsky D.I. The B-type lamin is required for somatic repression of testis-specific gene clusters. Proc. Natl. Acad. Sci. U.S.A. 2009; 106:3282–3287. 19218438 10.1073/pnas.0811933106 PMC2651240

[103] Cremer T. , Cremer M. Chromosome territories. Cold Spring Harb. Perspect. Biol. 2010; 2:a003889. 20300217 10.1101/cshperspect.a003889 PMC2829961

[104] Cook P.R. The organization of replication and transcription. Science. 1999; 284:1790–1795. 10364545 10.1126/science.284.5421.1790

[105] Razin S.V. , Gavrilov A.A., Pichugin A., Lipinski M., Iarovaia O.V., Vassetzky Y.S. Transcription factories in the context of the nuclear and genome organization. Nucleic Acids Res. 2011; 39:9085–9092. 21880598 10.1093/nar/gkr683 PMC3241665

[106] Papantonis A. , Cook P.R. Transcription factories: genome organization and gene regulation. Chem. Rev. 2013; 113:8683–8705. 23597155 10.1021/cr300513p

[107] Cenci G. , Bonaccorsi S., Pisano C., Verni F., Gatti M. Chromatin and microtubule organization during premeiotic, meiotic and early postmeiotic stages of *Drosophila**melanogaster* spermatogenesis. J. Cell Sci. 1994; 107:3521–3534. 7706403 10.1242/jcs.107.12.3521

[108] Sahut-Barnola I. , Pauli D. The *Drosophila* gene *stand still* encodes a germline chromatin-associated protein that controls the transcription of the ovarian tumor gene. Development. 1999; 126:1917–1926. 10101125 10.1242/dev.126.9.1917

[109] Wines D.R. , Talbert P.B., Clark D.V., Henikoff S. Introduction of a DNA methyltransferase into *Drosophila* to probe chromatin structure in vivo. Chromosoma. 1996; 104:332–340. 8575244 10.1007/BF00337221

[110] Aughey G.N. , Estacio Gomez A., Thomson J., Yin H., Southall T.D CATaDa reveals global remodelling of chromatin accessibility during stem cell differentiation in vivo. Elife. 2018; 7:e32341. 29481322 10.7554/eLife.32341 PMC5826290

[111] Gavrilov A.A. , Shevelyov Y.Y., Ulianov S.V., Khrameeva E.E., Kos P., Chertovich A., Razin S.V. Unraveling the mechanisms of chromatin fibril packaging. Nucleus. 2016; 7:319–324. 27249516 10.1080/19491034.2016.1190896 PMC4991243

[112] Van Bortle K. , Nichols M.H., Li L., Ong C.T., Takenaka N., Qin Z.S., Corces V.G. Insulator function and topological domain border strength scale with architectural protein occupancy. Genome Biol. 2014; 15:R82. 24981874 10.1186/gb-2014-15-5-r82 PMC4226948

[113] Jin F. , Li Y., Dixon J.R., Selvaraj S., Ye Z., Lee A.Y., Yen C.A., Schmitt A.D., Espinoza C.A., Ren B. A high-resolution map of the three-dimensional chromatin interactome in human cells. Nature. 2013; 503:290–294. 24141950 10.1038/nature12644 PMC3838900

[114] Le Dily F. , Baù D., Pohl A., Vicent G.P., Serra F., Soronellas D., Castellano G., Wright R.H., Ballare C., Filion G. et al. Distinct structural transitions of chromatin topological domains correlate with coordinated hormone-induced gene regulation. Genes Dev. 2014; 28:2151–2162. 25274727 10.1101/gad.241422.114 PMC4180976

[115] Comoglio F. , Park H.J., Schoenfelder S., Barozzi I., Bode D., Fraser P., Green A.R. Thrombopoietin signaling to chromatin elicits rapid and pervasive epigenome remodeling within poised chromatin architectures. Genome Res. 2018; 28:295–309. 10.1101/gr.227272.117 PMC5848609 29429976

[116] Bonev B. , Mendelson Cohen N., Szabo Q., Fritsch L., Papadopoulos G.L., Lubling Y., Xu X., Lv X., Hugnot J.P., Tanay A. et al. Multiscale 3D genome rewiring during mouse neural development. Cell. 2017; 171:557–572. 29053968 10.1016/j.cell.2017.09.043 PMC5651218

[117] Ing-Simmons E. , Vaid R., Bing X.Y., Levine M., Mannervik M., Vaquerizas J.M. Independence of chromatin conformation and gene regulation during *Drosophila* dorsoventral patterning. Nat. Genet. 2021; 53:487–499. 33795866 10.1038/s41588-021-00799-x PMC8035076

[118] Iwasaki Y.W. , Sriswasdi S., Kinugasa Y., Adachi J., Horikoshi Y., Shibuya A., Iwasaki W., Tashiro S., Tomonaga T., Siomi H. Piwi-piRNA complexes induce stepwise changes in nuclear architecture at target loci. EMBO J. 2021; 2:e108345. 10.15252/embj.2021108345 PMC8441340 34337769

[119] Boutanaev A.M. , Kalmykova A.I., Shevelyov Y.Y., Nurminsky D.I. Large clusters of co-expressed genes in the *Drosophila* genome. Nature. 2002; 420:666–669. 12478293 10.1038/nature01216

[120] Li H.B. , Ohno K., Gui H., Pirrotta V. Insulators target active genes to transcription factories and polycomb-repressed genes to polycomb bodies. PLos Genet. 2013; 9:e1003436. 23637616 10.1371/journal.pgen.1003436 PMC3630138

[121] Concia L. , Veluchamy A., Ramirez-Prado J.S., Martin-Ramirez A., Huang Y., Perez M., Domenichini S., Rodriguez Granados N.Y., Kim S., Blein T. et al. Wheat chromatin architecture is organized in genome territories and transcription factories. Genome Biol. 2020; 21:104. 32349780 10.1186/s13059-020-01998-1 PMC7189446

[122] Kind J. , Pagie L., de Vries S.S., Nahidiazar L., Dey S.S., Bienko M., Zhan Y., Lajoie B., de Graaf C.A., Amendola M. et al. Genome-wide maps of nuclear lamina interactions in single human cells. Cell. 2015; 163:134–147. 26365489 10.1016/j.cell.2015.08.040 PMC4583798

[123] Kind J. , Pagie L., Ortabozkoyun H., Boyle S., de Vries S.S., Janssen H., Amendola M., Nolen L.D., Bickmore W.A., van Steensel B. Single-cell dynamics of genome-nuclear lamina interactions. Cell. 2013; 153:178–192. 23523135 10.1016/j.cell.2013.02.028

[124] Corrales M. , Rosado A., Cortini R., van Arensbergen J., van Steensel B., Filion G.J. Clustering of *Drosophila* housekeeping promoters facilitates their expression. Genome Res. 2017; 27:1153–1161. 28420691 10.1101/gr.211433.116 PMC5495067

[125] Lee H. , Oliver B. Non-canonical *Drosophila* X chromosome dosage compensation and repressive topologically associated domains. Epigenetics Chromatin. 2018; 11:62. 30355339 10.1186/s13072-018-0232-y PMC6199721

[126] Schauer T. , Ghavi-Helm Y., Sexton T., Albig C., Regnard C., Cavalli G., Furlong E.E., Becker P.B. Chromosome topology guides the *Drosophila* dosage compensation complex for target gene activation. EMBO Rep. 2017; 18:1854–1868. 10.15252/embr.201744292 PMC5623837 28794204

[127] Ramírez F. , Lingg T., Toscano S., Lam K.C., Georgiev P., Chung H.R., Lajoie B.R., de Wit E., Zhan Y., de Laat W. et al. High-affinity sites form an interaction network to facilitate spreading of the MSL complex across the X chromosome in *Drosophil**a*. Mol. Cell. 2015; 60:146–162. 26431028 10.1016/j.molcel.2015.08.024 PMC4806858

