# Supplementary material for: Comparison of genome architecture at two stages of male germline cell differentiation in Drosophila
Source: Nucleic Acids Res. 2022 Feb 15;50(6):3203–25. doi: 10.1093/nar/gkac109 (PMC8989536; doi:10.1093/nar/gkac109)
Supplement: gkac109_Supplemental_Files [file gkac109_supplemental_files.zip › Supplementary Figures.pdf]

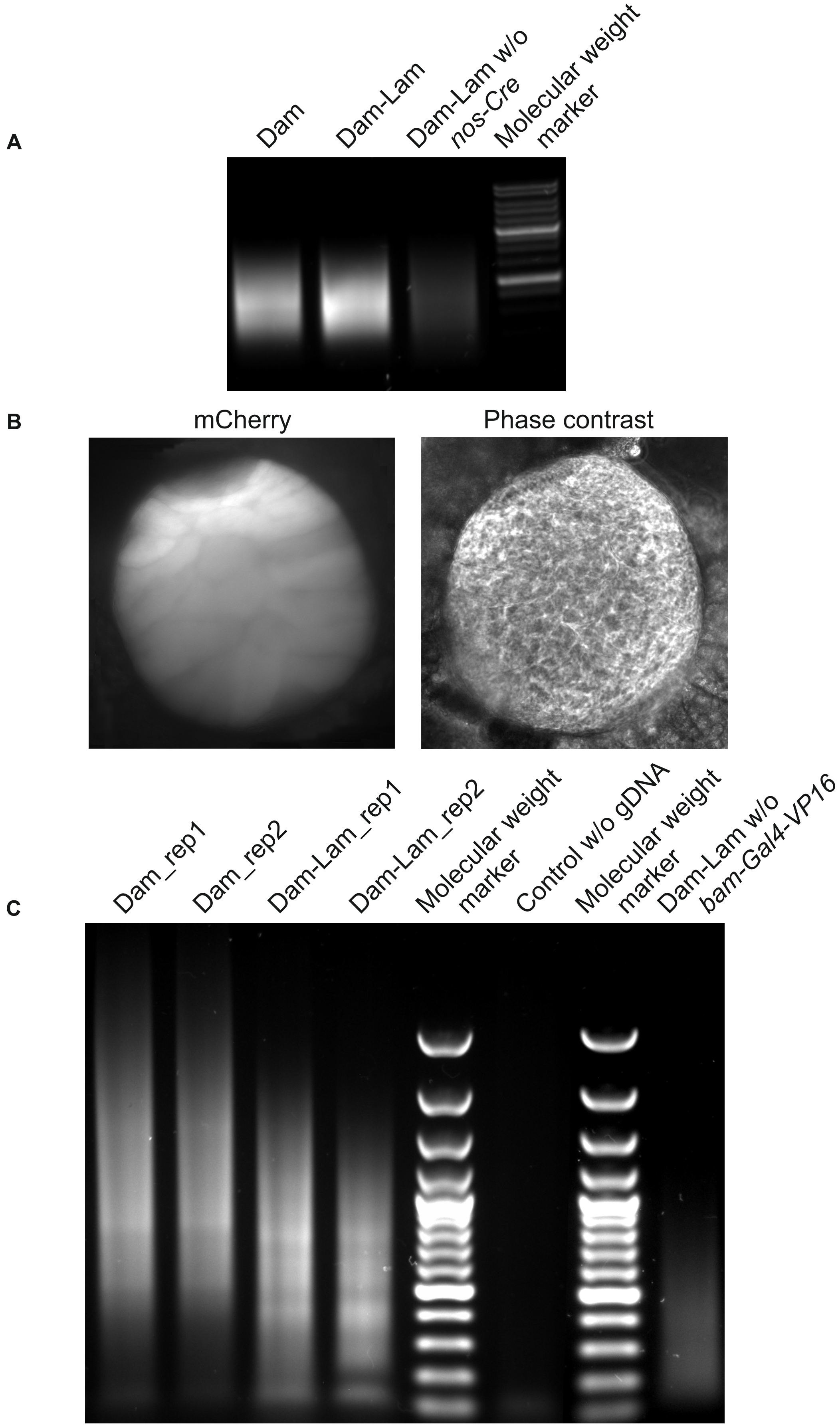

Figure S1. Lam-DamID in SpG and SpCs is highly specific. (**A**,**C**) Specific PCR-amplification of methylated DNA fragments in the germline cells. The characteristic smear of amplified fragments is drastically less pronounced in the samples without germline-specific *nos-Cre* (**A**) or *bam-Gal4-VP16* (**C**) drivers. (**B**) Imaging of larval testis of offspring after crossing males, carrying *pUAST-attB-LT3-Dam-Lam* construct (containing mCherry ORF upstream of Dam-Lam ORF), with *bam-Gal4-VP16* females. mCherry fluorescence is shown on the left panel, whereas phase contrast is shown on the right panel.

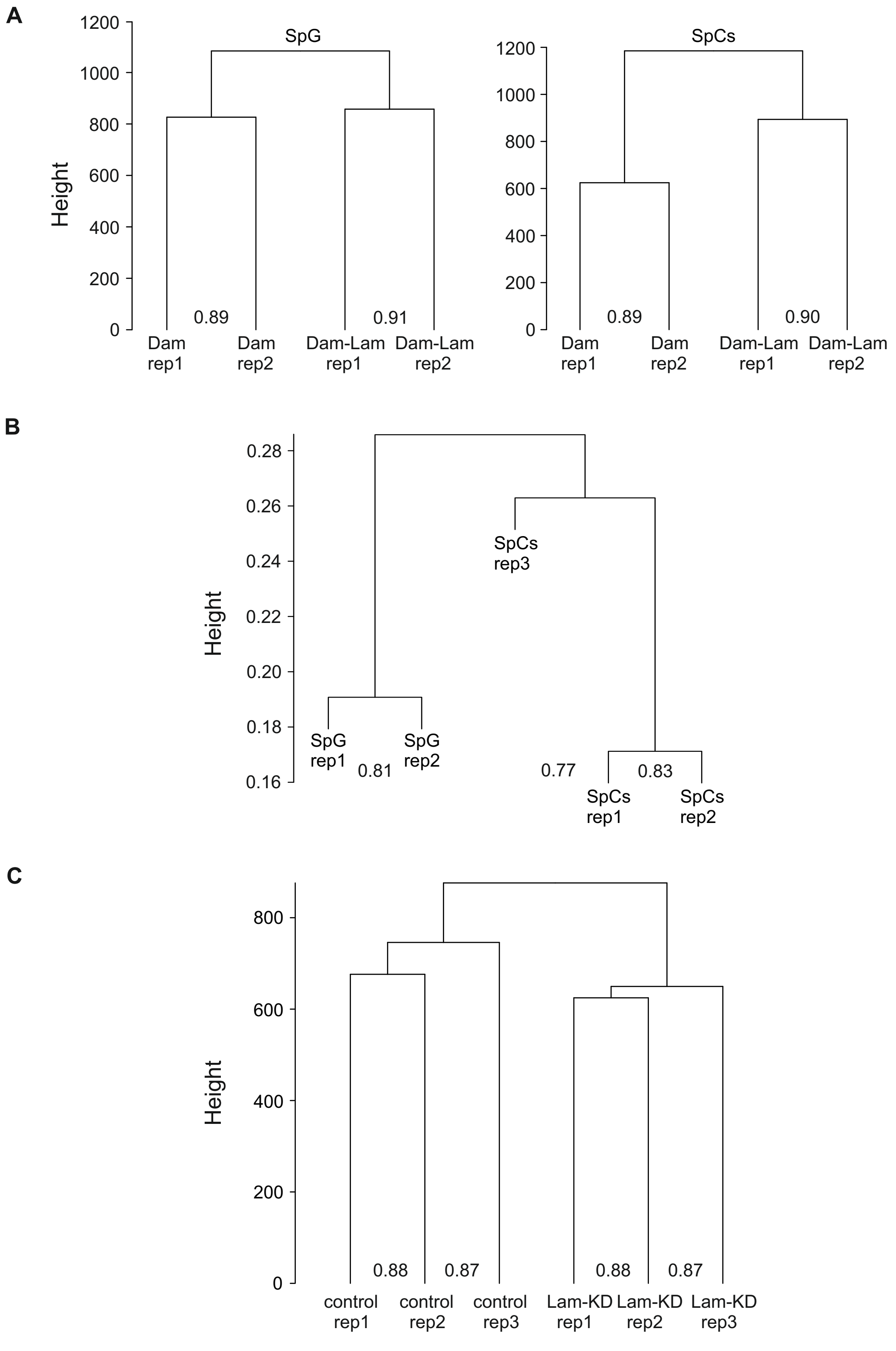

Figure S2. DamID, Hi-C and RNA-seq replicates are highly correlated. (**A**–**C**) Cluster analysis of biological replicates of DamID (**A**), Hi-C (**B**) and RNA-seq (**C**) experiments. Bin size - 0.3 kb (**A**), 10 kb (**B**), 0.5 kb (**C**). Spearman's (**A**,**C**) or stratum-adjusted (**B**) correlation coefficients for replicates are indicated.

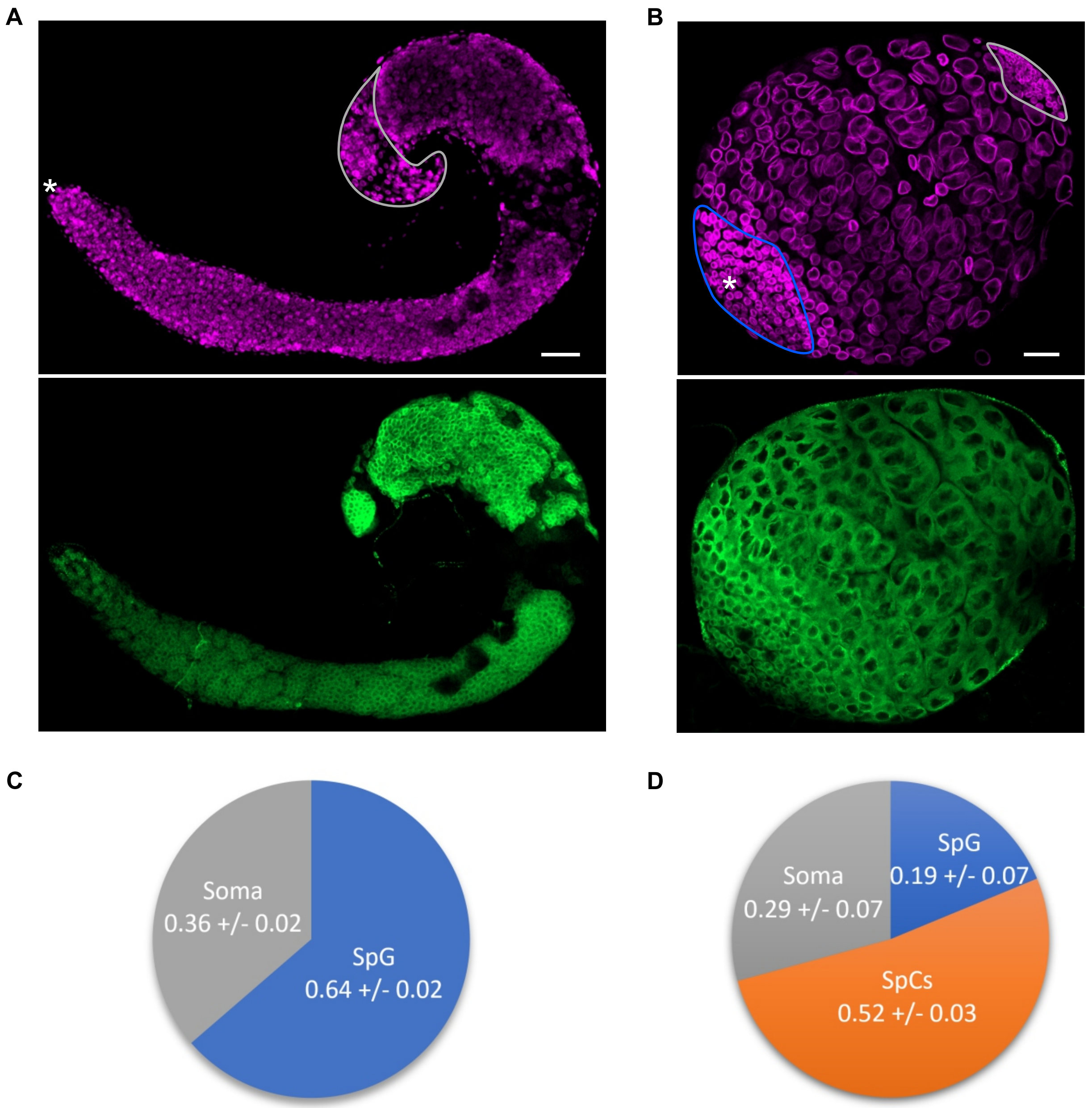

Figure S3. Estimation of the ratio of SpG, SpCs and somatic cells in *bam*$^{\Delta 86}$-mutant or 3rd instar larvae testes. (**A**,**B**) Confocal image of *bam*$^{\Delta 86}$-mutant (**A**) or 3rd instar larvae (**B**) testes stained with anti-Lam (violet) and anti-Vasa (green, marking germline cells) antibodies. Testis tip is marked by the asterisk. Zone mostly containing SpG is outlined by blue line (**B**). Zone of somatic cells at the opposite end of the testis is outlined by grey line (**A**,**B**). Scale bars are 50 μm (**A**) and 30 μm (**B**). (**C**,**D**) Quantitative evaluation of the number of different cell types in *bam*$^{\Delta 86}$-mutant or 3rd instar larvae testes. Three (**C**) or eight (**D**) testes were quantified.

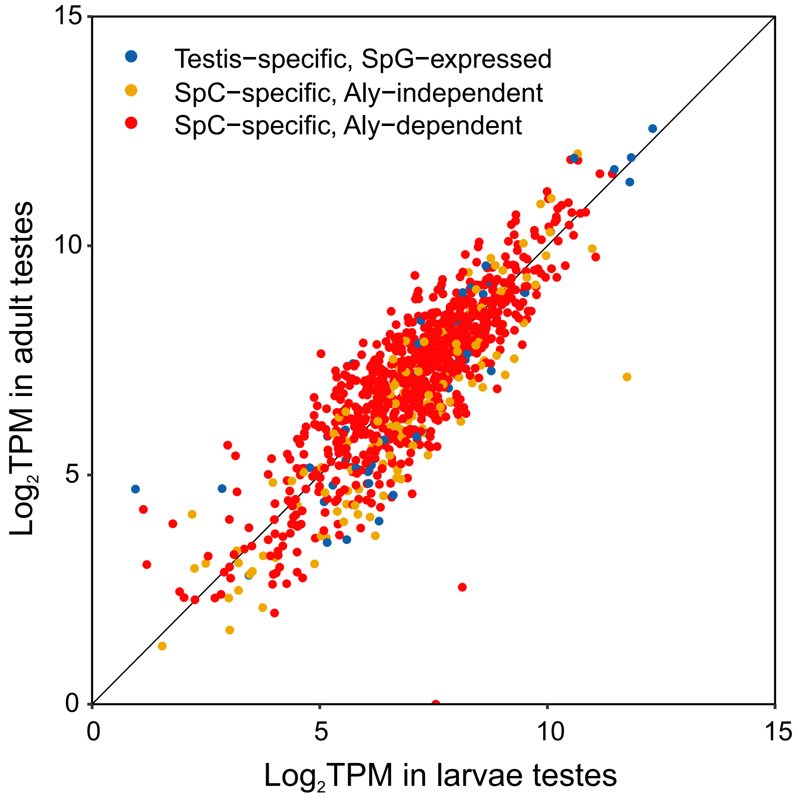

Figure S4. Testis-specific gene expression is highly correlated in testes isolated from third instar larvae or imago males. Scatter plot showing expression levels (in log_2TPM) of testis-specific genes in larvae testes (our RNA-seq data) or in imago testes (RNA-seq data from Laktionov *et al*. (86)). Dots corresponding to SpG-expressed genes are blue, corresponding to Aly-independent genes are orange, and corresponding to Aly-dependent genes are red.

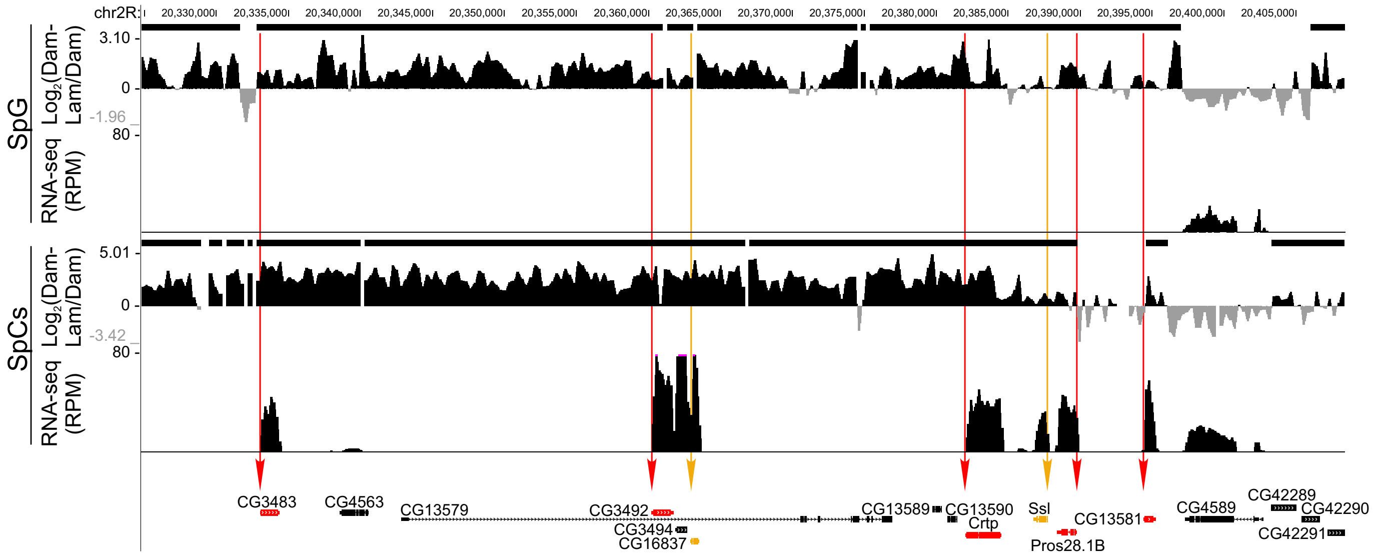

Figure S5. Aly-dependent, SpC-specific genes are mostly not revealed in the inter-LADs in SpCs. A screenshot of UCSC Genome Browser showing the $\log_2$(Dam-Lam/Dam) profiles as well as RNA-seq profiles (in RPM) in SpG and SpCs for the SpC-specific gene cluster from the *60D* genome region of the 2R chromosome. HMM-determined LADs are indicated by black rectangles over profiles. Arrows indicate promoter positions of Aly-dependent (red) and Aly-independent (orange) SpC-specific genes.

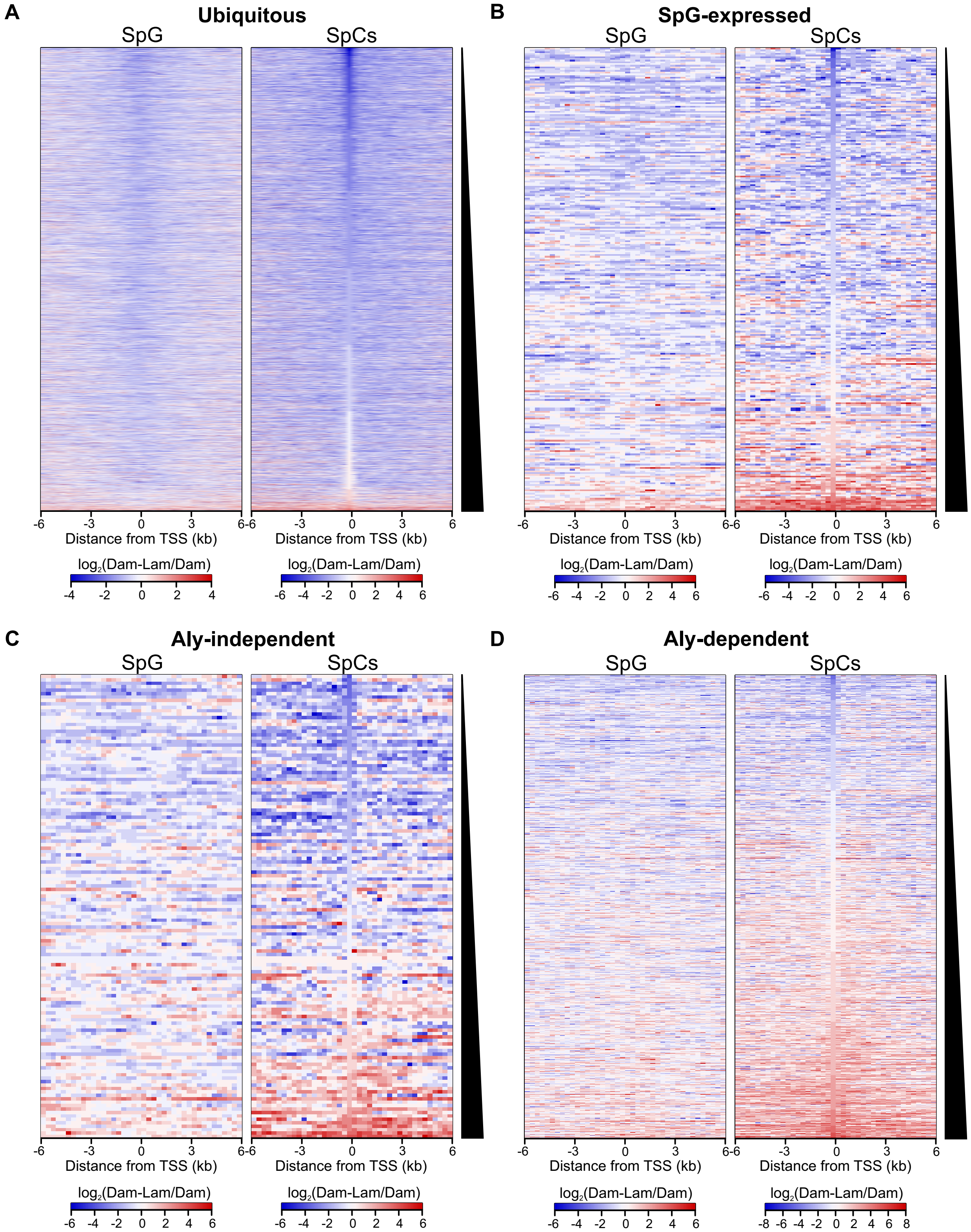

Figure S6. Heatmaps showing $\log_2$(Dam-Lam/Dam) profiles around promoters of different groups of genes. (**A**–**D**) $\log_2$(Dam-Lam/Dam) profiles around promoters of ubiquitous (**A**), SpG-expressed (**B**), Aly-independent (**C**) and Aly-dependent (**D**) genes in SpG (left panels) and SpCs (right panels). Profiles are sorted from low values (top) to high values (bottom) in SpCs.

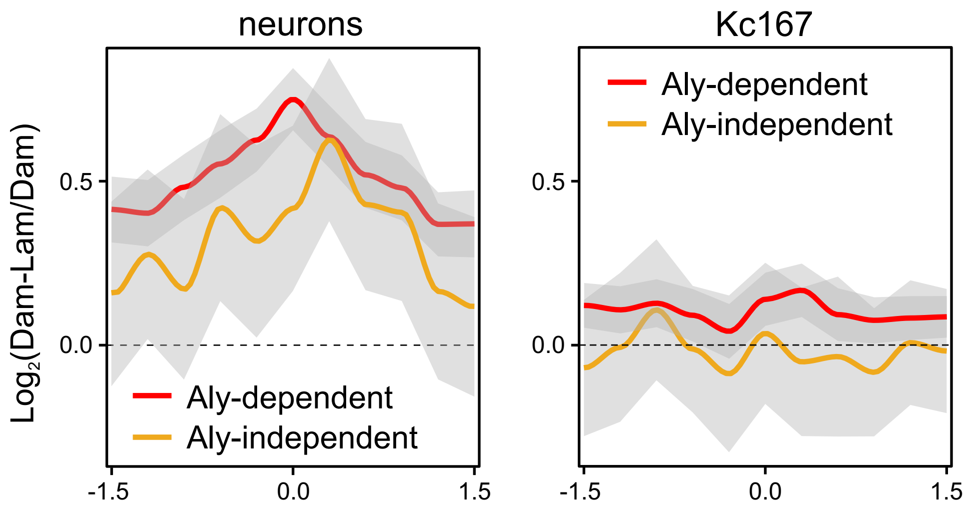

Figure S7. Aly-dependent and Aly-independent SpC-specific gene promoters are mostly localized in LADs in neurons and Kc167 cells. Averaged log_2(Dam-Lam/Dam) profiles around TSSs of Aly-dependent (red) and Aly-independent (orange) SpC-specific genes in neurons (left panel) or in Kc167 cells (right panel).

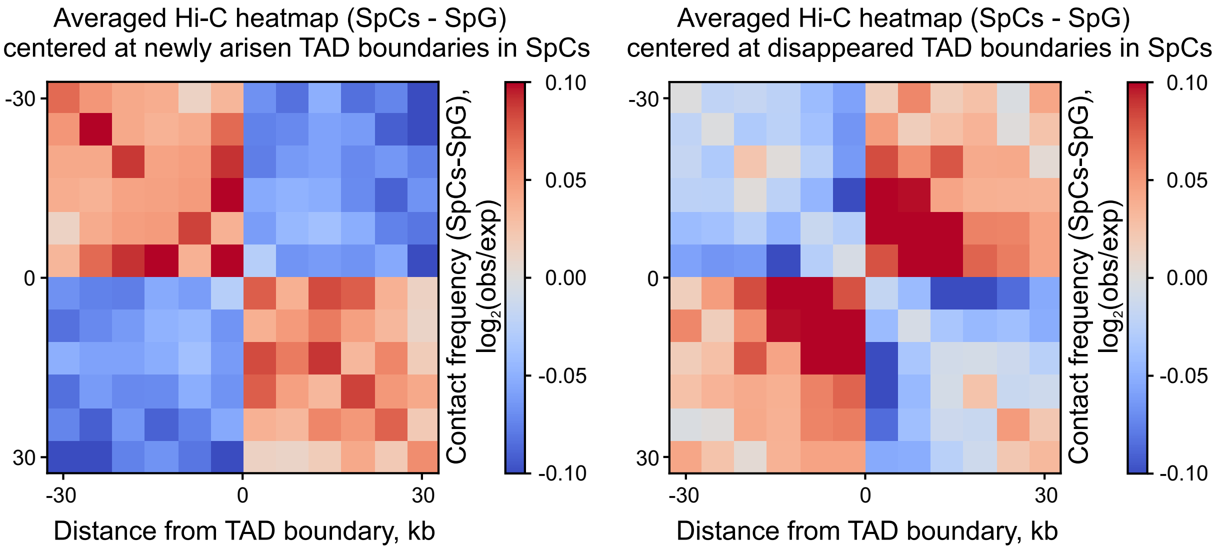

Figure S8. The appearance or disappearance of TAD boundaries in SpCs relative to SpG is not the result of errors of TAD calling algorithm. Averaged Hi-C heatmaps in SpC minus that in SpG around newly arisen (left panel) or disappeared (right panel) TAD boundaries in SpCs relative to SpG.

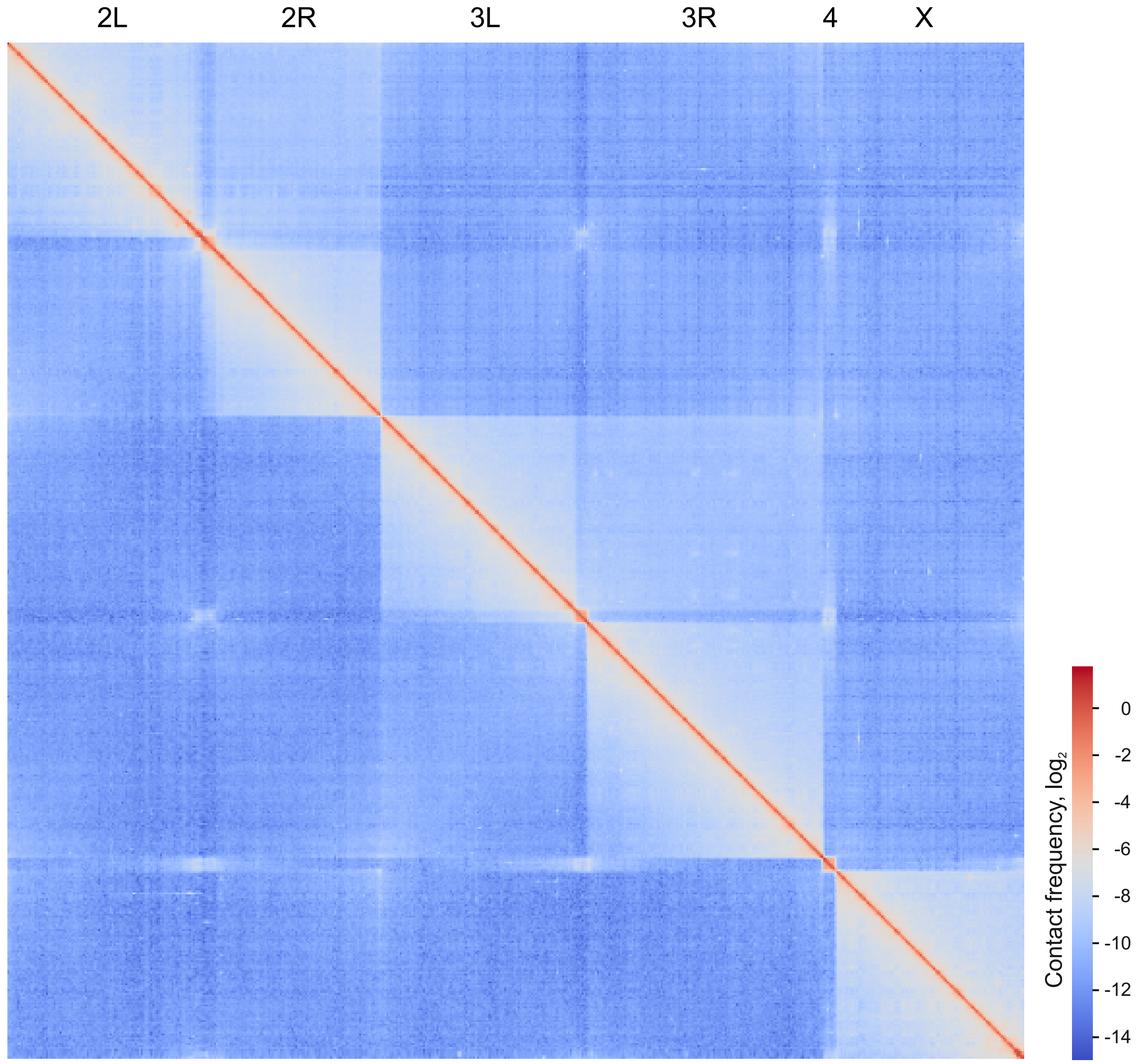

Figure S9. Whole-chromosome Hi-C heat maps in SpG (below diagonal) and SpCs (above diagonal).

	2L	2R	3L	3R	X
2L		1.40	0.44	0.46	0.45
2R	0.67		0.48	0.50	0.49
3L	0.35	0.38		1.25	0.46
3R	0.35	0.38	0.58		0.44
X	0.35	0.38	0.33	0.33	

Figure S10. Average Hi-C contact frequency ($\times 10^9$) of inter-arm contacts in SpG (below diagonal) and SpCs (above diagonal), normalized on the total number of contacts.

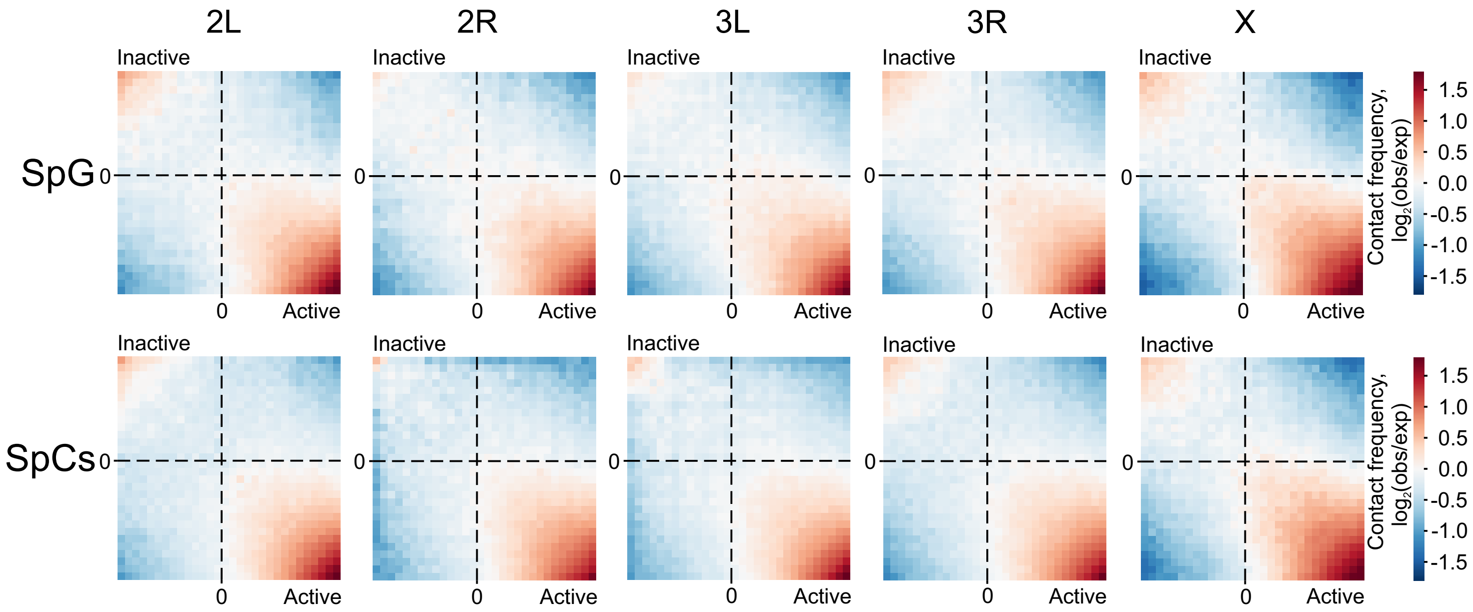

Figure S11. Saddle plots showing A and B compartments for individual chromosomes in SpG (upper panel) and SpCs (lower panel).

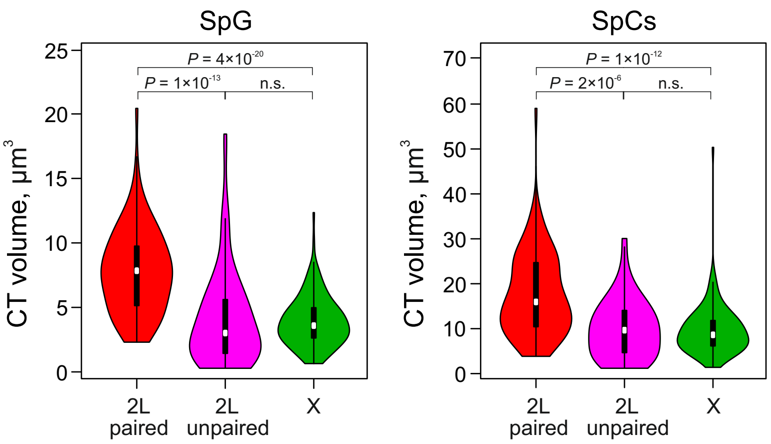

Figure S12. Violin plots showing distributions of volumes (roughly estimated in IMARIS) of the paired or unpaired 2L CTs or of the single X CTs in SpG (in *bam*$^{\Delta 86}$-mutant testes) and in SpCs (in WT testes). *P*-*v*alues were calculated using the M-W U-test. N.s. – non-significant.

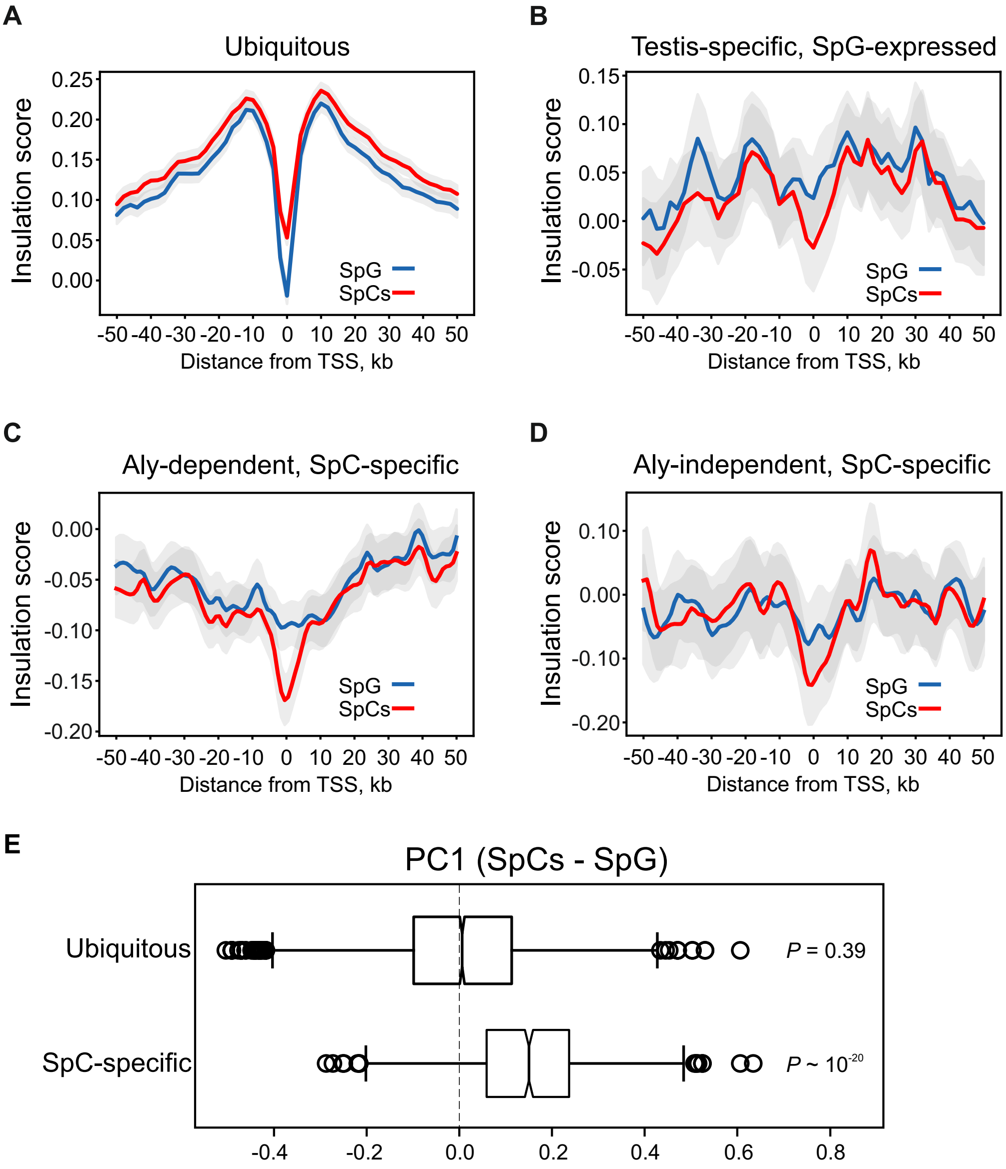

Figure S13. Ubiquitous, SpG-expressed and SpC-specific genes increase topological insulation of nearby regions. (**A**-**D**) Averaged IS profiles in SpG (blue curves) and SpCs (red curves) around the bin carrying promoters of ubiquitous (**A**), testis-specific, SpG-expressed (**B**), Aly-dependent, SpC-specific (**C**) and Aly-independent, SpC-specific (**D**) genes. The neighboring transcription was not filtered out. IS profiles were calculated for Hi-C heat maps built with 2-kb resolution using 12-kb sliding window. (**E**) The difference in PC1-values (SpCs - SpG) for 10-kb bins containing ubiquitous or SpC-specific gene TSSs. *P*-values were calculated using the M-W U-test.

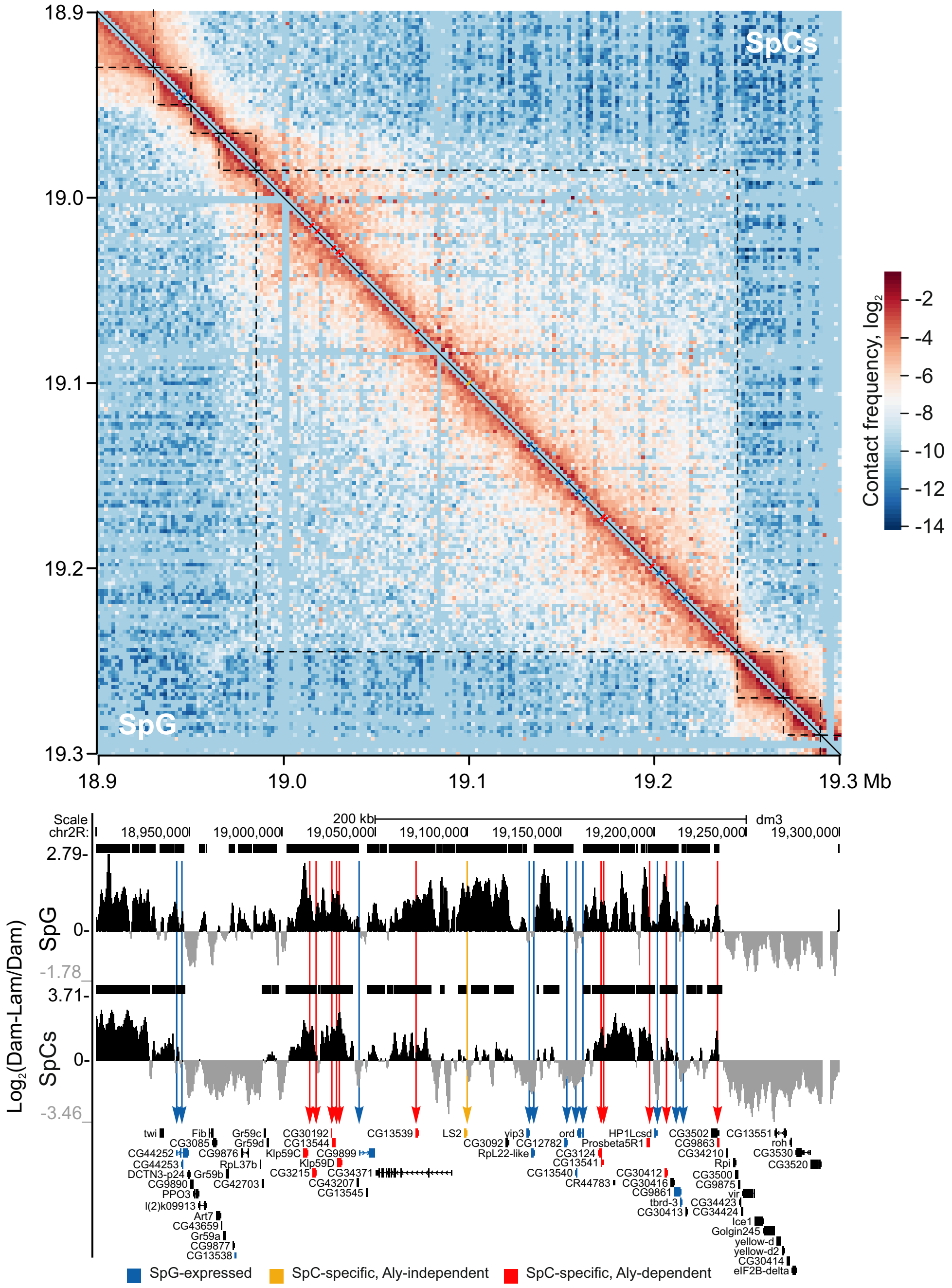

Figure S14. TAD boundaries do not correlate with the localization of SpC-specific genes. Hi-C heat map for the *59D* region of the 2R chromosome in SpG (below the diagonal) and SpCs (above the diagonal). TADs are demarcated by black dotted lines. Positions of testis-specific genes are shown by color rectangles at the diagonal. Below is the log_2(Dam-Lam/Dam) profiles in SpG and SpCs for the same region. LADs are indicated by black rectangles over profiles. Arrows indicate positions of promoters of testis-specific genes. Color code is given below.

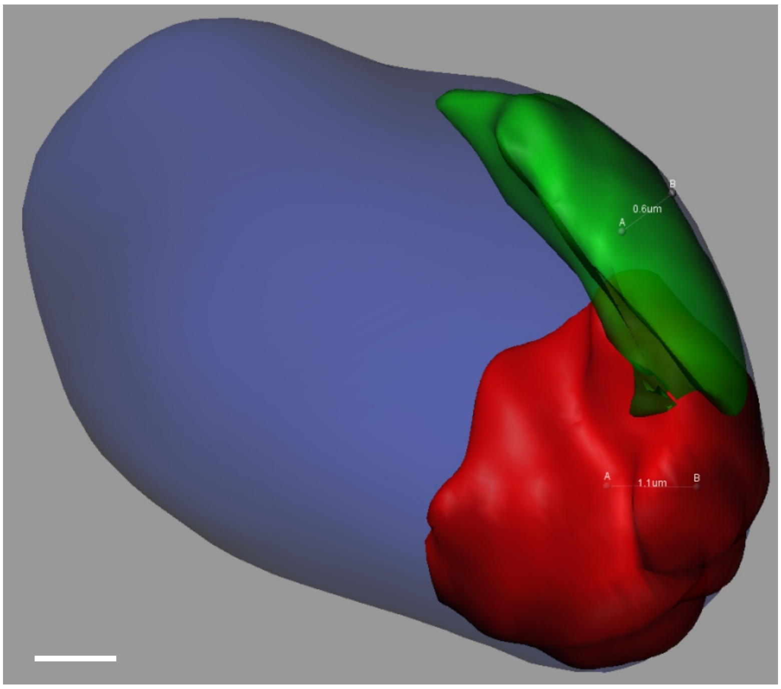

Figure S15. Reconstruction of chromosome territories in *IMARIS*. Scale bar 0.7 μm.

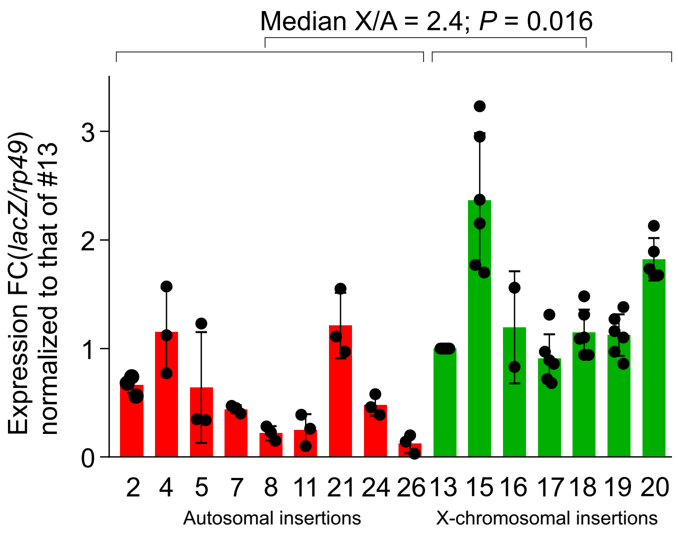

Figure S16. RT-qPCR analysis of *lacZ* expression in testes with autosomal or X-chromosomal insertions of *stil-lacZ* transgene. *rp49* expression was used for within sample normalization. Only lines carrying one dose of the transgene were compared. Autosomes carrying transgene were in the paired state. Relative expression of each transgene was normalized to the relative expression of line #13. Average values ±SD from two to six independent experiments are shown. M-W U-test was used for comparison of autosomal and X-chromosomal expression.
